# Function-Decoupled Environment-Gated Multimodal Fusion for Chlorophyll-*a* Retrieval in a Cold–Arid Shallow Lake

**DOI:** 10.3390/s26206362

**Published:** 2026-10-09

**Authors:** Huimin Li, Honghui Li, Xueliang Fu, Xiaohong Shi

**Affiliations:** 1 College of Computer and Information Engineering, Inner Mongolia Agricultural University, Hohhot 010018, China; lihuimin@imau.edu.cn (H.L.); lihh@imau.edu.cn (H.L.); 2 College of Water Conservancy and Civil Engineering, Inner Mongolia Agricultural University, Hohhot 010018, China

**Keywords:** Chlorophyll-*a* retrieval, multimodal sensor fusion, environment-gated fusion, Sentinel-1 SAR, Sentinel-2, shallow lakes

## Abstract

Lake Ulansuhai is a eutrophic shallow lake in the cold–arid region of northern China, where shallow water depth, frequent wind disturbance, and heterogeneous optical conditions complicate satellite-based chlorophyll-*a* (Chl-*a*) retrieval. To improve retrieval under such complex observation conditions, this study develops a function-decoupled environment-gated fusion framework (FDEGF) that integrates optical, synthetic aperture radar (SAR), and meteorological observations through predefined computational roles. Sentinel-2 optical features form the direct retrieval pathway, Sentinel-1 SAR features provide auxiliary information, and ERA5 meteorological variables condition the participation of the auxiliary pathway at the sampling-event level. A satellite–ground matched dataset containing 287 valid observations from 2017 to 2019 was constructed for Lake Ulansuhai. Across 31 paired random train–test splits, FDEGF showed a generally positive absolute-error advantage relative to simplified fusion structures, reducing median MAE by 0.360–$0.467\;\mu g\;L^{-1}$. Further analyses showed that model performance depended on the correspondence between event-level meteorological gating and the SAR auxiliary pathway, while the three input pathways exhibited differentiated model dependence. Under unseen-station validation, FDEGF retained positive spatial transferability within the lake. However, leave-one-sampling-event-out validation yielded a pooled $R^{2}$ of −0.144 and no consistent advantage over the structural controls. These results indicate that explicitly separating direct retrieval, auxiliary information, and meteorological gating provides a structurally explicit strategy for multimodal Chl-*a* retrieval within the represented sampling-event domain, while transfer to previously unseen sampling events remains limited.

## 1. Introduction

Lake Ulansuhai is a typical shallow eutrophic lake in the cold–arid region of northern China and an important receiving water body of the Hetao Irrigation District. Long-term nutrient inputs, shallow water depth, frequent wind disturbance, and pronounced environmental variability have contributed to persistent eutrophication and complex water-quality conditions [1,2]. Continuous monitoring of the lake is therefore important for understanding its ecological status and supporting water-environment management. Chlorophyll-*a* (Chl-*a*), as an important indicator of phytoplankton biomass and eutrophication status, is widely used to characterize lake trophic conditions. However, conventional field sampling is limited in temporal frequency and spatial coverage. Satellite remote sensing provides an important means of extending Chl-*a* observations over larger spatial areas and repeated observation periods.

Substantial progress has been made in satellite-based Chl-*a* retrieval. Spectral approaches exploit the characteristic response of chlorophyll in the red and red-edge regions and have shown good applicability in eutrophic and optically complex waters [3,4,5,6]. Data-driven approaches can further capture nonlinear relationships between remotely sensed observations and Chl-*a* concentration and have been successfully applied to different inland waters and satellite sensors [7,8,9,10,11,12]. Recent multi-lake studies have also demonstrated the potential of Sentinel-2 for extending Chl-*a* monitoring across geographically diverse inland-water systems [13]. Together, these studies provide a strong methodological basis for satellite-based Chl-*a* retrieval.

Nevertheless, retrieval relationships developed under one set of water and observation conditions cannot necessarily be transferred directly to Lake Ulansuhai. In shallow lakes, wind-induced sediment resuspension can alter suspended-material concentrations and water-leaving reflectance, while variations in bottom influence, suspended particles, and optical water types can further modify the relationship between spectral observations and Chl-*a* [14,15,16,17,18]. Changes in optical water types and particle-dominated conditions have also been associated with different Chl-*a* retrieval behavior under changing environmental conditions [19]. These findings indicate that the optical relationship with Chl-*a* may be condition-dependent rather than invariant. This issue is particularly relevant to Lake Ulansuhai because its shallow depth and frequent disturbance create heterogeneous observation conditions. Consequently, optical observations remain fundamental to Chl-*a* retrieval, but the stability of an optical retrieval relationship may vary across observation conditions.

Complementary sensing information provides a potential way to characterize part of this variability. Sentinel-1 synthetic aperture radar (SAR) is sensitive to surface-scattering characteristics and can respond to changes in water-surface conditions, thereby providing information that is not directly represented by optical reflectance [20,21,22]. The joint use of Sentinel-1 and Sentinel-2 has also shown potential for lake Chl-*a* retrieval, suggesting that SAR observations can provide supplementary information to optical measurements [23]. Meanwhile, meteorological reanalysis products such as ERA5 describe the meteorological background associated with satellite observations [24]. Optical, SAR, and meteorological observations therefore offer potentially complementary descriptions of water-color response, radar scattering, and meteorological background, respectively.

The key challenge is not merely whether these heterogeneous observations can be combined, but how they should participate in the retrieval process. Direct feature concatenation can learn nonlinear interactions among multiple information sources, but it does not explicitly encode their different observational roles or reveal whether their contributions should vary with observation context. Recent multimodal remote-sensing studies have explored modality-specific branches, gated fusion, and dynamic weighting to improve the integration of heterogeneous observations [25,26,27]. Other studies have further emphasized the importance of interpretable multimodal fusion and understanding the contributions of individual information sources [28,29]. These developments demonstrate the value of explicitly structured multimodal integration, but the structured use of heterogeneous observations remains particularly important for shallow-lake Chl-*a* retrieval, where different sensing sources do not necessarily provide the same type of information and their contributions may vary with observation context.

To address this issue, this study develops a multimodal fusion framework for Chl-*a* retrieval in Lake Ulansuhai. The framework adopts a function-decoupled environment-gated fusion architecture (FDEGF), in which heterogeneous observations are assigned predefined computational roles according to their observational characteristics. Sentinel-2 optical features are assigned to the direct retrieval pathway, whereas Sentinel-1 SAR features are assigned to an auxiliary pathway. ERA5-derived wind speed and air temperature form a meteorological gating pathway that conditions the participation of the auxiliary pathway at the sampling-event level. The final retrieval therefore combines a direct optical estimate with an event-conditioned auxiliary contribution rather than relying on unrestricted fusion of all input variables.

A satellite–ground matched dataset containing 287 valid observations from 17 sampling events during 2017–2019 was constructed by integrating field-measured Chl-*a* with Sentinel-2 optical observations, Sentinel-1 SAR observations, and ERA5 meteorological information. Based on this dataset, the proposed framework was evaluated from multiple perspectives, including overall retrieval performance and error characteristics, structural contribution, temporal-matching sensitivity, pathway dependence, within-lake spatial transferability, and event-independent transfer. Rather than treating overall accuracy as the sole criterion, this multilevel evaluation was designed to distinguish structural behavior within the represented sampling-event domain from within-lake transfer to unseen stations and transfer to previously unseen sampling events.

The main contributions of this study are summarized as follows:

1. A multimodal satellite–ground matched dataset was constructed for Lake Ulansuhai by integrating field-measured Chl-*a*, Sentinel-2 optical observations, Sentinel-1 SAR observations, and ERA5 meteorological information. The dataset contains 287 valid observations from 17 sampling events and provides a basis for investigating Chl-*a* retrieval under heterogeneous observation conditions in a cold–arid shallow lake.
2. A function-decoupled environment-gated multimodal fusion framework was developed to assign heterogeneous observations distinct computational roles in the retrieval process. The architecture assigns optical, SAR, and meteorological inputs to direct, auxiliary, and event-conditioned gating pathways, respectively, thereby making the information flow among modalities explicit.
3. A multilevel validation strategy was established to distinguish within-domain retrieval behavior, within-lake spatial transfer, and event-independent transfer. Repeated random partitions, temporal-matching sensitivity analysis, structural diagnostics, unseen-station validation, and leave-one-sampling-event-out validation were combined to clarify both the structural behavior and the current generalization boundary of FDEGF.

## 2. Study Area and Multimodal Data

### 2.1. Study Area

**Figure 1:**
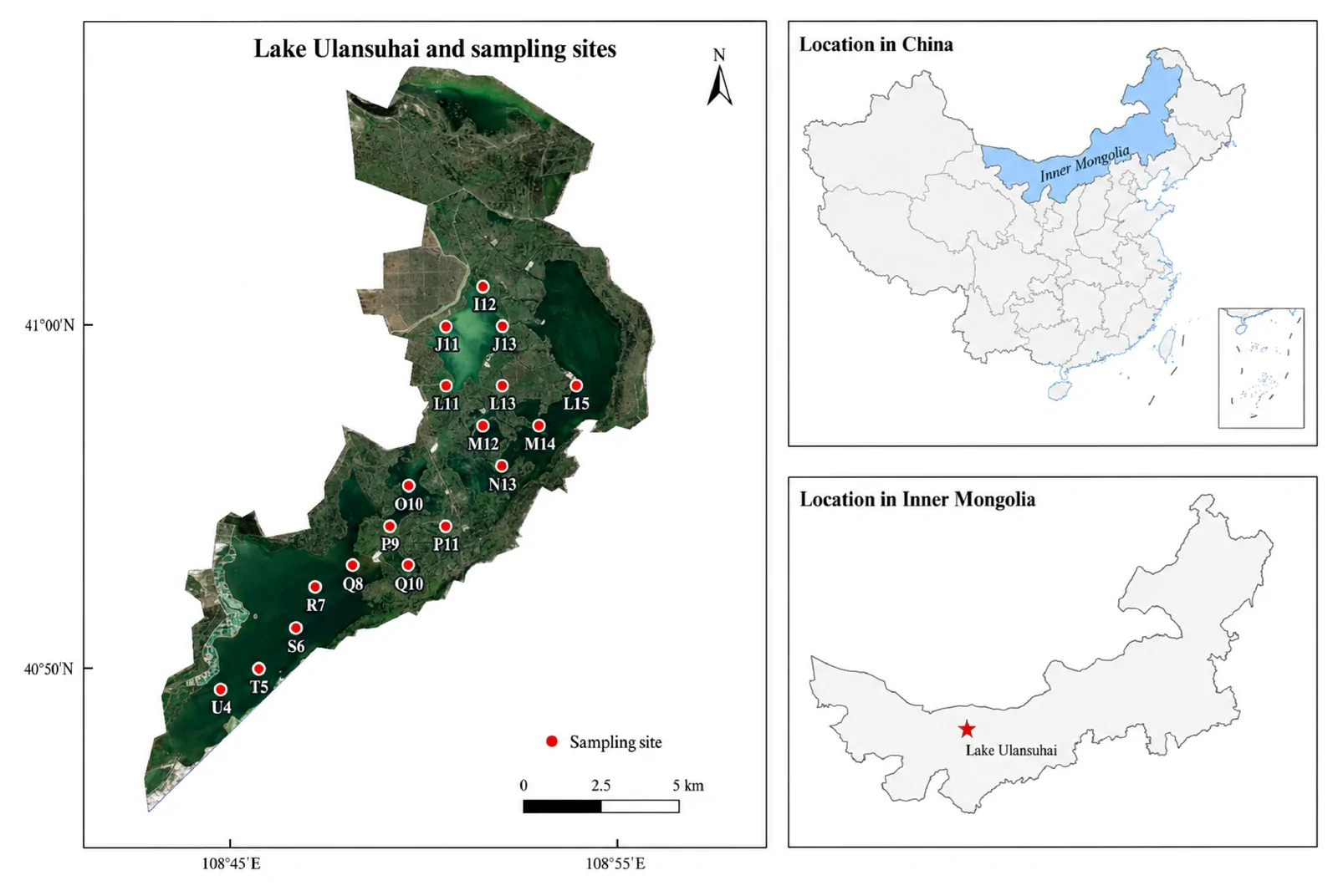
Location of Lake Ulansuhai and distribution of sampling stations during 2017–2019. The gray dashed lines in the “Location in China” inset indicate provincial-level administrative boundaries.

Lake Ulansuhai is located in Bayannur City, Inner Mongolia Autonomous Region, China, at the eastern end of the Hetao Irrigation District. It extends approximately from $40{}^{\circ}36^{\prime }$ to $41{}^{\circ}03^{\prime }\;N$ and from $108{}^{\circ}43^{\prime }$ to $108{}^{\circ}57^{\prime }\;E$. The lake is a typical shallow wetland in the cold–arid region of northwestern China and serves as the principal receiving water body for agricultural drainage from the Hetao Irrigation District. Its average water depth is approximately 1.3m. Shallow water, strong evaporation, frequent wind disturbance, and pronounced seasonal variability contribute to heterogeneous optical and observation conditions. The study area and sampling stations during 2017–2019 are shown in Figure 1.

Lake Ulansuhai receives long-term inputs of nitrogen, phosphorus, and other nutrients from agricultural drainage, and eutrophication remains pronounced [1,30,31,32]. Its hydrological and water-quality conditions are jointly influenced by regional climate, irrigation and drainage activities, and internal biogeochemical processes. These characteristics make Lake Ulansuhai a suitable study area for investigating multimodal Chl-*a* retrieval under shallow, wind-disturbed, and optically heterogeneous conditions.

### 2.2. Multimodal Data Acquisition and Preprocessing

**Figure 2:**
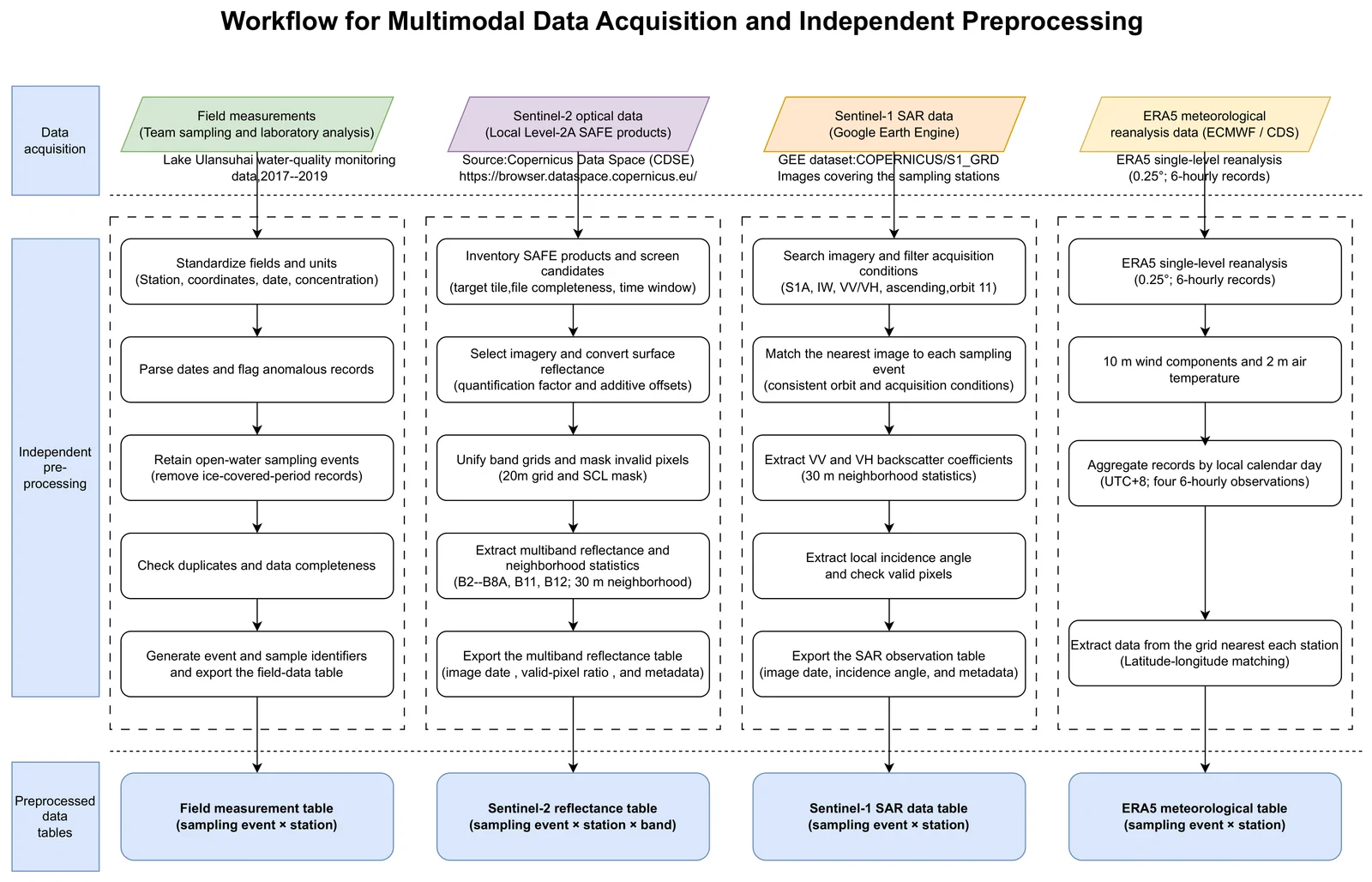
Workflow for multimodal data acquisition and preprocessing. Dashed boxes indicate the independent preprocessing workflows for the four data sources, while horizontal dashed lines separate the data acquisition, preprocessing, and output-table stages.

The dataset integrates field observations collected during 2017–2019, Sentinel-2 optical observations, Sentinel-1 synthetic aperture radar (SAR) observations, and ERA5 meteorological reanalysis data. The overall data-acquisition, preprocessing, and satellite–ground matching workflow is illustrated in Figure 2.

#### 2.2.1. In Situ Measurements

Field sampling was conducted at Lake Ulansuhai from 2017 to 2019. Eighteen stations were sampled in 2017, and 20 stations were sampled in both 2018 and 2019. Surface-water samples were collected at approximately 0.5m below the water surface, stored under refrigerated and dark conditions, and transported to the laboratory within 24h in accordance with HJ 493–2009 [33].

Chl-*a* concentration was measured using acetone extraction and ultraviolet spectrophotometry. Filter membranes were extracted in 90% acetone for 24h under dark and low-temperature conditions. Parallel measurements were averaged when the relative standard deviation was below 10%; otherwise, the analysis was repeated. Only records from open-water periods were retained because winter ice cover substantially changes both water optical properties and satellite observation conditions.

Station names, coordinates, sampling dates, and concentration units were standardized, and incomplete or repeated records were removed. Each record was assigned an event identifier (event_id) and a sample identifier (sample_id) for subsequent multimodal matching.

Four observations in the finalized field-data table were recorded as $0.0\;\mu g\;L^{-1}$. Sample-specific original laboratory records and corresponding analytical detection or quantification limits were not available for retrospective verification. These observations were therefore retained as recorded and were not reinterpreted as either true zero concentrations or below-detection-limit measurements. A zero-exclusion sensitivity analysis was additionally conducted to assess whether these records affected the main retrieval and structural conclusions.

#### 2.2.2. Sentinel-2 Optical Data

Sentinel-2 Level-2A Multispectral Instrument (MSI) products were downloaded from the Copernicus Data Space Ecosystem [34], primarily for tile T49TCF. Candidate images were searched within 10d before and after each field-sampling date, and the complete image acquired closest to the corresponding field event was selected.

The Level-2A products used in this study provide bottom-of-atmosphere reflectance generated through the standard Sen2Cor processing chain (version 2.10.03; processing baseline 05.00). Reflectance values were reconstructed from the stored digital numbers using the product-specific quantification factor and band-specific offset:

(1) $$R_{\lambda }=\frac{DN_{\lambda }+O_{\lambda }}{Q},$$

where $R_{\lambda }$ denotes surface reflectance, $DN_{\lambda }$ is the digital number, $O_{\lambda }$ is the additive offset, and *Q* is the reflectance quantification factor.

All bands were aligned to the 20m B5 grid. Reflectance bands were resampled using bilinear interpolation, whereas the scene classification layer (SCL) was resampled using nearest-neighbor interpolation. Pixels affected by no data, saturation, cloud, cloud shadow, cirrus, snow, or ice were excluded. The retained pixels were not restricted to the SCL water class because dense algal blooms and highly turbid water can be assigned to other SCL categories.

A 30m circular neighborhood was established around each sampling station. Median reflectance and valid-pixel proportion were extracted for the available bands. The satellite acquisition date and its absolute temporal difference from the corresponding field-sampling date were also recorded. Records without valid observations in the key optical bands were excluded.

ACOLITE or another aquatic-specific atmospheric-correction processor was not tested in the present study. Sen2Cor Level-2A products were used as a consistent preprocessing source, but the absence of concurrent in situ water-surface reflectance prevented independent validation of alternative atmospheric-correction processors. Residual atmospheric and adjacency effects may therefore remain, and the optical features are interpreted within the adopted Level-2A processing chain.

#### 2.2.3. Sentinel-1 SAR Data

Sentinel-1 observations were obtained from the COPERNICUS/S1_GRD collection in Google Earth Engine. Sentinel-1A images acquired in interferometric wide-swath (IW) mode with VV and VH polarization, ascending orbit, and relative orbit number 11 were selected to constrain acquisition geometry and maintain a consistent observation configuration.

For each sampling event, the nearest acquisition satisfying these conditions was selected. Matches with absolute temporal differences of up to 3d were treated as the core temporal range, whereas acquisitions with differences of 4–5 d were retained within the extended matching range. The final dataset also contained two temporally more distant Sentinel-1 matches in 2019. For one sampling campaign in August 2019, 19 samples were matched to a Sentinel-1 acquisition with an absolute temporal difference of 6d. For one sampling campaign in May 2019, no valid acquisition was available within the routine matching range, and the nearest valid Sentinel-1A acquisition satisfying the same orbit constraints was retained as a supplementary match for 16 samples with an absolute temporal difference of 12d. These extended matches and their temporal offsets were retained in the dataset metadata for traceability.

VV and VH backscatter coefficients were extracted within a 30m circular neighborhood at a 10m scale. Neighborhood medians were retained to reduce the influence of geolocation differences and isolated anomalous pixels. The corresponding acquisition date, orbit information, and local incidence angle were also preserved for each matched observation. Records without valid VV or VH observations were excluded.

Although the acquisition configuration was constrained by using the same platform, imaging mode, orbit direction, and relative orbit, incidence angle still ranged from approximately 35.064° to 36.421° over the final matched records. Incidence angle was therefore retained as diagnostic metadata rather than used as a model predictor, and its association with VV and VH was examined separately.

#### 2.2.4. ERA5 Meteorological Data

Meteorological information was derived from ERA5 single-level reanalysis data [24]. The reconstruction used a 0.25° spatial grid and a 6-hourly temporal subset containing the 10m eastward and northward wind components and 2m air temperature.

For each field-sampling date, the reanalysis grid cell nearest to Lake Ulansuhai was identified, and the four 6-hourly observations corresponding to the local calendar day (UTC+8) were aggregated. Wind speed was first calculated from the eastward and northward wind components at each time step, and the local-day mean wind speed was retained as the meteorological wind feature. The local-day mean 2m air temperature was retained as the temperature feature and converted from Kelvin to degrees Celsius. Records with incomplete meteorological information were excluded.

Because the ERA5 variables were aggregated at the local-calendar-day scale and the spatial resolution was coarse relative to the lake, all stations within the same sampling event received identical wind-speed and air-temperature values. The 17 sampling events therefore corresponded to 17 unique wind-speed–temperature combinations, with no within-event variation. These variables are treated as coarse event-level meteorological descriptors rather than station-specific environmental states.

### 2.3. Multimodal Matching and Feature Construction

#### 2.3.1. Spatiotemporal Matching

The in situ observation table was used as the reference for multimodal matching. Sentinel-2, Sentinel-1, and ERA5-derived meteorological records were linked to the field observations using event_id and station name. The uniqueness of this composite key was verified before merging.

Sampling dates, station coordinates, satellite acquisition dates, absolute temporal differences, orbit information, and Sentinel-1 incidence-angle metadata were retained to preserve the provenance of each record. Unmatched or inconsistent records were excluded. The annual datasets from 2017, 2018, and 2019 were subsequently combined into a unified candidate dataset. The retained temporal offsets were used in the subsequent temporal-matching sensitivity analysis.

#### 2.3.2. Feature Construction

Two features were retained from each information source to limit model dimensionality while preserving clear observational meaning. For the optical modality, Sentinel-2 B4 and B5 reflectance were used to calculate the normalized-difference chlorophyll index (NDCI) and the B5/B4 reflectance ratio:

(2) $$\mathrm{NDCI}=\frac{R_{B5}-R_{B4}}{R_{B5}+R_{B4}},$$

and

(3) $${\mathrm{Ratio}}_{B5/B4}=\frac{R_{B5}}{R_{B4}}.$$

The neighborhood medians of VV and VH backscatter were retained as the SAR features. For each ERA5 time step, wind speed was calculated from the eastward and northward wind components as follows:

(4) $$u_{10}=\sqrt{u^{2}+v^{2}},$$

where *u* and *v* denote the 10m eastward and northward wind components, respectively. The resulting wind-speed values were averaged over the four 6-hourly records corresponding to each local calendar day. The local-day mean wind speed and local-day mean 2m air temperature were retained as the two meteorological features.

The three feature groups were represented as follows:

(5) $$X_{\mathrm{opt}}={\left[\mathrm{NDCI},{\mathrm{Ratio}}_{B5/B4}\right]}^{T},$$

(6) $$X_{\mathrm{sar}}={\left[\sigma_{\mathrm{VV}}^{0,\mathrm{dB}},\sigma_{\mathrm{VH}}^{0,\mathrm{dB}}\right]}^{T},$$

and

(7) $$X_{\mathrm{met}}={\left[u_{10},t_{2m}\right]}^{T}.$$

These three feature groups were assigned to the direct, auxiliary, and event-conditioned gating pathways, respectively, in the proposed FDEGF architecture, as described in Section 3.

### 2.4. Dataset Construction and Characteristics

The initial candidate dataset contained 348 records. A valid sample required a field-measured Chl-*a* concentration and complete finite values for NDCI, B5/B4, VV, VH, $u_{10}$, and $t_{2m}$. After preprocessing and sample screening, 287 records from 17 sampling events were retained and 61 were excluded, corresponding to a retention rate of 82.5%. The final dataset contained 103 samples from 2017 and 92 samples from each of 2018 and 2019. Year, station, sampling event, satellite acquisition date, temporal difference, orbit information, and matching metadata were retained together with the six model input features.

Chl-*a* concentrations ranged from 0.00 to $87.35\;\mu g\;L^{-1}$, with a mean of $11.40\;\mu g\;L^{-1}$ and a standard deviation of $12.57\;\mu g\;L^{-1}$. Four observations were recorded as $0.0\;\mu g\;L^{-1}$, as described in Section 2.2. Twenty-seven samples had concentrations of at least $30\;\mu g\;L^{-1}$, accounting for 9.4% of the final dataset.

**Figure 3:**
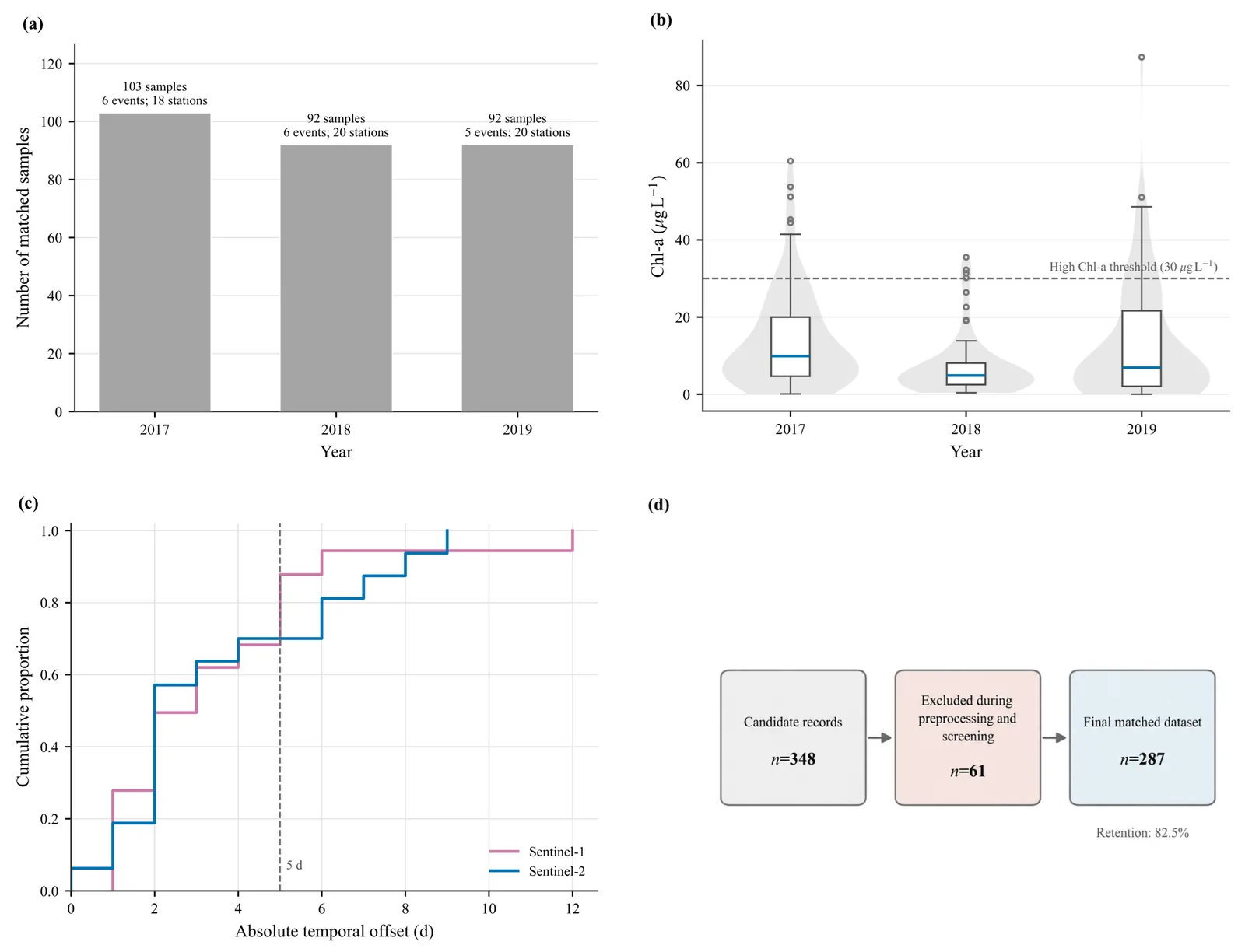
Composition and preprocessing characteristics of the multimodal satellite–ground matched dataset for Lake Ulansuhai during 2017–2019: (**a**) annual sample coverage; (**b**) annual Chl-*a* distributions (gray shading: distribution density; boxes: interquartile ranges; blue lines: medians; whiskers: non-outlier ranges; open circles: outliers; dashed line: high-concentration threshold of $30\;\mu g\;L^{-1}$); (**c**) absolute satellite–ground temporal differences; and (**d**) dataset construction and sample screening.

The annual sample composition, Chl-*a* distribution, satellite–ground temporal differences, and sample-screening process are summarized in Figure 3.

## 3. Methodology

This section describes the proposed multimodal fusion framework for Chl-*a* retrieval in Lake Ulansuhai. The problem formulation, function-decoupled environment-gated fusion framework (FDEGF), model training strategy, experimental validation design, evaluation metrics, and statistical analyses are presented.

### 3.1. Problem Formulation

Chlorophyll-*a* (Chl-*a*) retrieval is formulated as a supervised continuous regression problem using heterogeneous optical, SAR, and meteorological observations.

After satellite–ground matching, preprocessing, and sample screening, the dataset is expressed as follows:

(8) $$D={\left\{(X^{\left(i\right)},y^{\left(i\right)})\right\}}_{i=1}^{N},\;N=287,$$

where $X^{\left(i\right)}\in R^{6}$ represents the multisource feature vector of the *i*th sample and $y^{\left(i\right)}$ denotes the corresponding measured Chl-*a* concentration.

The input features are separated into three modality-specific groups:

(9) $$X={\left[X_{\mathrm{opt}}^{T},X_{\mathrm{sar}}^{T},X_{\mathrm{met}}^{T}\right]}^{T},$$

where

(10) $$X_{\mathrm{opt}}={\left[\mathrm{NDCI},{\mathrm{Ratio}}_{B5/B4}\right]}^{T},$$

(11) $$X_{\mathrm{met}}={\left[u_{10},t_{2m}\right]}^{T},$$

(12) $$X_{\mathrm{sar}}={\left[\sigma_{\mathrm{VV}}^{0,\mathrm{dB}},\sigma_{\mathrm{VH}}^{0,\mathrm{dB}}\right]}^{T}.$$

Rather than treating the three feature groups as interchangeable inputs, FDEGF assigns them distinct computational roles: optical features form the direct retrieval pathway, SAR features form the auxiliary pathway, and the meteorological features condition the auxiliary pathway at the sampling-event level.

### 3.2. Function-Decoupled Environment-Gated Fusion Framework

The term function-decoupled refers to the explicit assignment of different computational roles to heterogeneous observations rather than unrestricted feature fusion. The proposed function-decoupled environment-gated fusion framework (FDEGF) contains three pathways: a direct retrieval pathway, an auxiliary pathway, and a meteorological gating pathway. Optical, SAR, and meteorological inputs are assigned to these pathways according to their roles in the fusion architecture.

#### 3.2.1. Overall Framework

**Figure 4:**
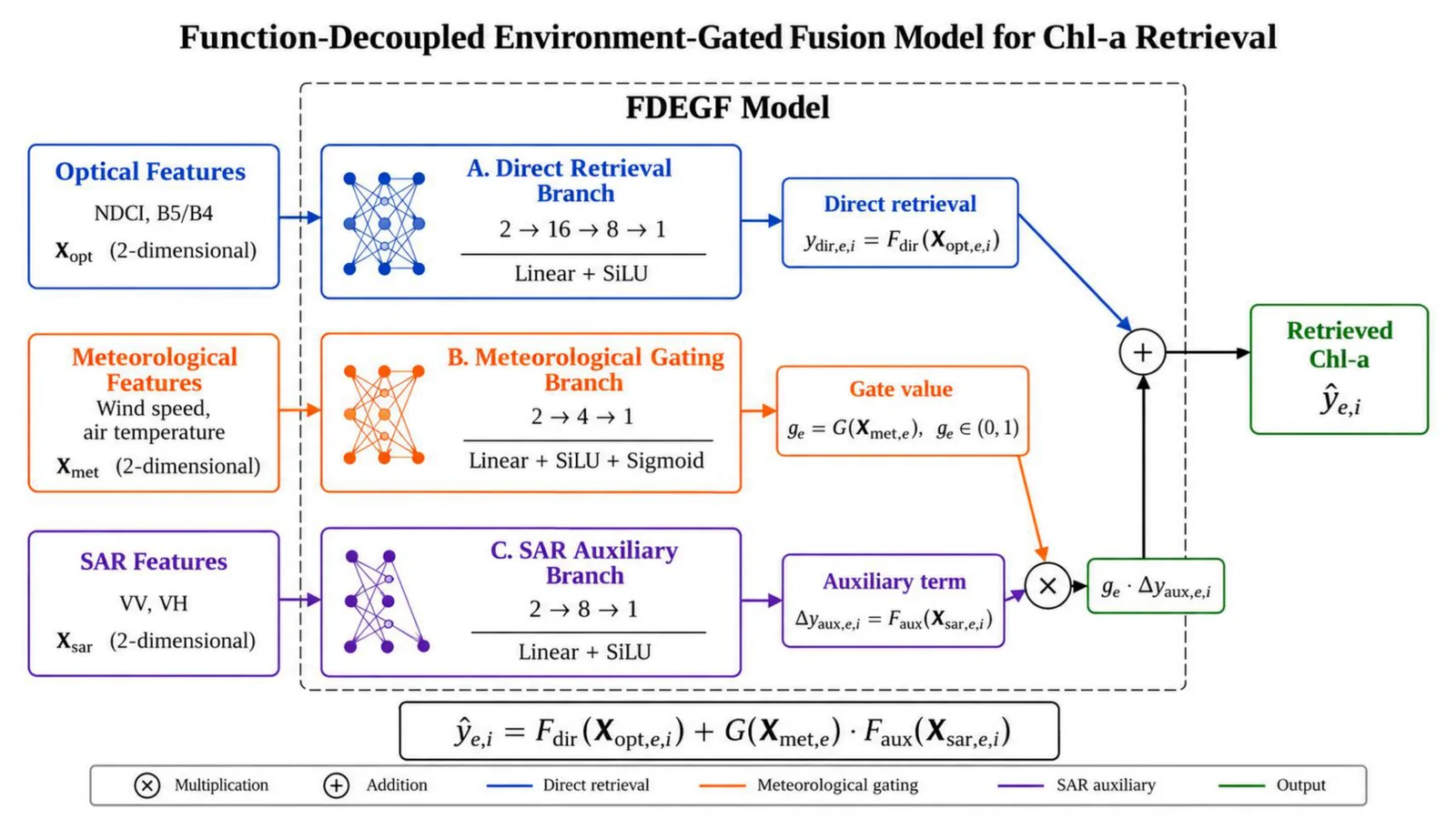
Overall architecture of the function-decoupled environment-gated fusion model.

The architecture of FDEGF is shown in Figure 4. The optical pathway provides the direct Chl-*a* retrieval output, the SAR pathway provides an auxiliary contribution, and the meteorological pathway controls the participation of the auxiliary pathway at the sampling-event level.

For station *i* within sampling event *e*, the three pathway outputs are defined as follows:

(13) $$y_{dir,e,i}=F_{dir}\left(X_{opt,e,i}\right),$$

(14) $$\Delta y_{aux,e,i}=F_{aux}\left(X_{sar,e,i}\right),$$

and

(15) $$g_{e}=G\left(X_{met,e}\right),\;g_{e}\in \left(0,1\right).$$

The final retrieval is as follows:

(16) $${\hat{y}}_{e,i}=y_{dir,e,i}+g_{e}\Delta y_{aux,e,i},$$

or equivalently:

(17) $${\hat{y}}_{e,i}=F_{dir}\left(X_{opt,e,i}\right)+G\left(X_{met,e}\right)F_{aux}\left(X_{sar,e,i}\right).$$

This formulation preserves an explicit primary–auxiliary information flow: the optical pathway provides the direct retrieval, while the contribution of the SAR pathway is modulated by the meteorological context of each sampling event. Although the gate is shared by stations within the same event, the effective auxiliary contribution remains station-dependent because the SAR output varies among stations. The pathway roles are defined by the architecture and should not be interpreted as a unique physical decomposition of the retrieval process. The pathway mappings are nonlinear neural-network functions rather than fixed-order polynomial functions; therefore, no polynomial order is imposed.

#### 3.2.2. Optical Retrieval Pathway

The optical pathway takes

$$X_{\mathrm{opt}}={\left[\mathrm{NDCI},{\mathrm{Ratio}}_{B5/B4}\right]}^{T}$$

as input and uses a multilayer perceptron with architecture 2→16→8→1.

The hidden layers use the SiLU activation function, while the output layer uses a linear activation function. The output represents the direct Chl-*a* retrieval defined by the optical pathway.

#### 3.2.3. SAR Auxiliary Pathway

The SAR pathway receives

$$X_{\mathrm{sar}}={\left[VV,VH\right]}^{T}$$

and uses a multilayer perceptron with architecture 2→8→1.

The output $\Delta y_{aux}$ is not an independent Chl-*a* estimate, but an auxiliary term learned from the SAR observations. It can take either positive or negative values and contributes to the final retrieval only through the gated fusion operation.

#### 3.2.4. Meteorological Gating Pathway

The gating pathway uses

$$X_{\mathrm{met}}={\left[u_{10},t_{2m}\right]}^{T}$$

and adopts a multilayer perceptron with architecture 2→4→1.

The output is transformed using the sigmoid function:

(18) $$Sigmoid\left(z\right)=\frac{1}{1+\exp(-z)},$$

which constrains the gate value to the interval (0,1). A larger gate value allows a greater contribution from the SAR auxiliary pathway, whereas a smaller value suppresses it. Because the meteorological inputs are shared by stations within the same sampling event, the resulting gate is event-conditioned rather than station-specific.

### 3.3. Model Training

#### 3.3.1. Data Standardization

Before each training procedure, all input features were standardized using statistics calculated exclusively from the corresponding training set:

(19) $${\tilde{x}}_{ij}=\frac{x_{ij}-\mu_{j}^{train}}{\sigma_{j}^{train}},$$

where $\mu_{j}^{train}$ and $\sigma_{j}^{train}$ denote the mean and standard deviation of feature *j* calculated from the training samples. The target Chl-*a* concentration was standardized using the same strategy:

(20) $${\tilde{y}}_{i}=\frac{y_{i}-\mu_{y}^{train}}{\sigma_{y}^{train}}.$$

The test samples were transformed using only the corresponding training-set parameters. Standardization parameters were recalculated whenever the training composition changed, thereby preventing information leakage across random, station-grouped, and event-grouped evaluations.

#### 3.3.2. Loss Function and Parameter Optimization

The three branches of FDEGF were jointly trained in an end-to-end manner. On the standardized scale, the model output was calculated as follows:

(21) $$\begin{array}{cc} {\hat{\tilde{y}}}^{\left(i\right)}= & F_{\mathrm{dir}}\left({\tilde{X}}_{\mathrm{opt}}^{\left(i\right)}\right)\\ \; & +G\left({\tilde{X}}_{\mathrm{met}}^{\left(e(i)\right)}\right)F_{\mathrm{aux}}\left({\tilde{X}}_{\mathrm{sar}}^{\left(i\right)}\right), \end{array}$$

where e(i) denotes the sampling event associated with sample *i*. Samples from the same event therefore share the same standardized meteorological input to the gating pathway.

The training objective was the mean squared error (MSE):

(22) $$L_{MSE}=\frac{1}{N_{train}}\sum_{i=1}^{N_{train}}{\left({\tilde{y}}^{\left(i\right)}-{\hat{\tilde{y}}}^{\left(i\right)}\right)}^{2}.$$

No branch-specific supervision was imposed; all three pathways were updated through the final fused retrieval error. The pathway outputs should therefore be interpreted as architecture-defined computational components rather than independently identified physical quantities.

The model parameters were jointly optimized using Adam with a learning rate of 0.005 for 501 epochs. Full-batch training was adopted considering the limited dataset size. After inference, standardized outputs were transformed back to the original Chl-*a* concentration scale:

(23) $${\hat{y}}^{\left(i\right)}={\hat{\tilde{y}}}^{\left(i\right)}\sigma_{y}^{train}+\mu_{y}^{train}.$$

#### 3.3.3. Training Procedure of FDEGF

The complete training and inference workflow of FDEGF is summarized in Algorithm 1. For each data partition, all standardization parameters are estimated exclusively from the training set, and the three pathways are jointly optimized using the final fused retrieval error.
**Algorithm 1** Training and Inference Workflow of FDEGF  1:**Input:** training set $D_{tr}$, test set $D_{te}$, learning rate η=0.005, number of epochs E=501  2:Compute feature and target statistics using $D_{tr}$ only  3:Standardize the training and test data using the training-set statistics  4:Initialize the parameters of the direct retrieval, auxiliary, and meteorological gating pathways  5:**for** k=1 to *E* **do**  6:   Compute the direct retrieval output:$$y_{\mathrm{dir}}=F_{\mathrm{dir}}\left({\tilde{X}}_{\mathrm{opt}}\right)$$  7:   Compute the auxiliary output:$$\Delta y_{\mathrm{aux}}=F_{\mathrm{aux}}\left({\tilde{X}}_{\mathrm{sar}}\right)$$  8:   Compute the event-conditioned gate value:$$g=G({\tilde{X}}_{\mathrm{met}})$$  9:   Compute the gated auxiliary contribution:$$g\Delta y_{\mathrm{aux}}$$10:   Fuse the direct and auxiliary pathways:$$\hat{\tilde{y}}=y_{\mathrm{dir}}+g\Delta y_{\mathrm{aux}}$$11:   Calculate the mean squared error (MSE) loss12:   Update all trainable parameters using Adam13:**end for**14:Apply the trained FDEGF model to the standardized test data15:Transform the standardized retrieval outputs back to the original Chl-*a* concentration scale16:**Output:** retrieved Chl-*a* concentrations

#### 3.3.4. Implementation

The model was implemented in Python (version 3.13.9) using Torch (version 2.7.1+cu118). Conventional machine learning baselines were implemented using scikit-learn (version 1.7.2). The XGBoost baseline was implemented using the xgboost package (version 3.2.0). Random states were fixed in each experiment to improve reproducibility.

### 3.4. Experimental Design

Experiments were designed to assess retrieval performance and the specific contribution of the function-decoupled gated architecture. Repeated random partitions were used to characterize resampling stability, while structural diagnostics, sensitivity analyses, and grouped validation were used to examine pathway dependence, robustness to data and matching choices, and transfer to unseen stations and sampling events.

#### 3.4.1. Optical Retrieval Across Wind-Speed Groups

The 287 samples were divided into low-, medium-, and high-wind-speed groups using the one-third and two-third quantiles of the complete wind-speed distribution. The same thresholds were used throughout the related descriptive analyses. This grouping was used only for post hoc comparison and was not involved in model training or gate construction.

Sample size, wind-speed range, air temperature, and Chl-*a* distribution were summarized for each group. Relationships between the optical features (NDCI and B5/B4) and measured Chl-*a*, together with Optical-only retrieval errors, were compared across the three groups. Because wind speed was shared by stations within the same sampling event, the groups were treated as coarse descriptive categories rather than independent sample-specific environmental states.

#### 3.4.2. Retrieval-Model Performance Comparison

Retrieval performance under repeated random partitioning was evaluated using 31 paired 80:20 train–test splits with random states 0–29 and 42. For each partition, all compared models used exactly the same training and test samples.

FDEGF was compared with Ridge regression, support vector regression (SVR), random forest (RF), XGBoost, and multilayer perceptron (MLP). All models were evaluated using identical test samples and the same retrieval metrics.

Because the 31 partitions reuse the same 287 observations and have substantially overlapping training and test sets, they were interpreted as resampling-based stability summaries rather than independent experimental replicates. Model differences were summarized using paired metric differences, their distributions, and better–worse counts across the repeated partitions.

#### 3.4.3. Structural Ablation Experiment

Four independently trained structures were compared to evaluate the contribution of the main components of FDEGF:

(24) $${\hat{y}}_{e,i}^{Opt}=d_{e,i},$$

(25) $${\hat{y}}_{e,i}^{NG}=d_{e,i}+a_{e,i},$$

(26) $${\hat{y}}_{e,i}^{CG}=d_{e,i}+\bar{g}a_{e,i},$$

and

(27) $${\hat{y}}_{e,i}^{FDEGF}=d_{e,i}+g_{e}a_{e,i}.$$

Here, $d_{e,i}$ denotes the direct retrieval output, $a_{e,i}$ the auxiliary output, $g_{e}$ the event-conditioned gate, and $\bar{g}$ a learnable constant gate shared across all samples.

The Optical-only structure retains only the direct optical pathway. The No-gate structure introduces the auxiliary pathway without gating, whereas the Constant-gate structure tests whether event-conditioned gating provides additional benefit beyond global scaling. All structures were independently initialized and trained using the same 31 paired random train–test partitions.

#### 3.4.4. Structural Diagnostics of Multimodal Fusion

Structural diagnostics were conducted to examine how the three information pathways contributed to the learned fusion structure. These analyses were used to characterize architecture-dependent model behavior rather than to assign unique physical meanings to individual branch outputs.

##### Branch-Output and Sample-Level Diagnostics

For each out-of-sample (OOS) prediction, the direct output $d_{e,i}$, auxiliary output $a_{e,i}$, event-conditioned gate $g_{e}$, and gated auxiliary contribution

(28) $$c_{e,i}=g_{e}a_{e,i}$$

were recorded. The sample-level absolute-error benefit of FDEGF relative to a comparison model *m* was defined as follows:

(29) $$B_{e,i}^{\left(m\right)}=|y_{e,i}-{\hat{y}}_{e,i}^{\left(m\right)}\left|-\right|y_{e,i}-{\hat{y}}_{e,i}^{FDEGF}|.$$

A positive value indicates a lower absolute retrieval error for FDEGF. The comparison models included the independently trained Optical-only and No-gate structures. For samples appearing in multiple random partitions, repeated OOS results were summarized by their median before sample-level analysis.

##### Initialization-Sensitivity Analysis

To examine the stability of the learned decomposition, FDEGF was retrained on the original single random partition (random state 42) using 20 independent network initializations, with the architecture, preprocessing, optimizer, learning rate, and number of epochs unchanged. Cross-initialization variation and pairwise agreement were evaluated for the final prediction, direct output, auxiliary output, gate, and gated auxiliary contribution. This analysis distinguishes stability of the final retrieval from identifiability of the internal branch decomposition.

##### Event-Constrained Gate-Mismatch Experiment

Because the meteorological gate is shared within each sampling event, three gate-mismatch schemes were evaluated while keeping the direct and auxiliary outputs unchanged. The original sample-wise global reassignment was retained as a reference. Complete event-level gate blocks were then reassigned among sampling events, and a further scheme restricted event-level reassignment to events from the same season. Seasonal groups were defined by sampling month as spring (March–May), summer (June–August), autumn (September–November), and winter (December–February).

For the event-level perturbation,

(30) $${\hat{y}}_{e,i}^{mis}=d_{e,i}+g_{\pi (e)}a_{e,i},$$

where π(e) denotes a permutation of sampling events. Each perturbation scheme was repeated 100 times for every random partition. Performance degradation was interpreted as model dependence on the correspondence between event-conditioned meteorological gating and the auxiliary pathway, rather than as evidence of physical causality.

##### Grouped Input-Feature Permutation

The optical, meteorological, and SAR feature groups were independently permuted, with variables within the same group sharing a common permutation to preserve within-group relationships. Global, within-year, and within-season schemes were evaluated. Each permutation was repeated 30 times for every random partition and feature group. Performance changes were interpreted as pathway-dependence diagnostics rather than direct measures of physical causality.

##### SAR Geometry–Wind–Residual Diagnostics

Associations of VV and VH with Sentinel-1 incidence angle, near-contemporaneous ERA5 wind, and independent Optical-only OOS retrieval residuals were examined to evaluate the interpretation of the SAR auxiliary pathway. The Optical-only residual for each sample was defined as

(31) $$r_{e,i}^{opt}=y_{e,i}-\mathrm{median}\left({\hat{y}}_{e,i,\mathrm{OOS}}^{Opt}\right),$$

using only predictions obtained when that observation was held out of training. Correlation and partial-correlation analyses were conducted before and after controlling for incidence angle, with event-cluster bootstrap intervals used to account for the sampling-event structure. These variables were used only for diagnostics and were not added to the FDEGF inputs.

##### Branch Behavior Across Wind-Speed Groups

Gate values, auxiliary contributions, and sample-level benefits were also summarized across the three wind-speed groups defined above.

#### 3.4.5. Sensitivity Analyses

Temporal-matching sensitivity was evaluated by progressively restricting the satellite–ground temporal offsets. Five subsets were considered: the full dataset (N=287, 17 events); exclusion of the 12-d Sentinel-1 event, retaining Sentinel-1 offsets within 6 d (N=271, 16 events); Sentinel-1 offsets within 5 d (N=252, 15 events); joint Sentinel-1 and Sentinel-2 offsets within 5 d (N=166, 10 events); and joint offsets within 3 d (N=110, 7 events). FDEGF and the three structural controls were evaluated under the same paired random-partition protocol for each subset. Differences in absolute performance among subsets were not interpreted as causal effects of temporal matching because event composition and target distributions also changed as the matching criteria were tightened.

A second sensitivity analysis examined the four observations recorded as $0.0\;\mu g\;L^{-1}$. These records were excluded without changing the train–test membership of the remaining observations, and retrieval performance was compared on the common nonzero test samples.

#### 3.4.6. Grouped Transfer Validation

##### Unseen-Station Validation

Five-fold cross-validation grouped by sampling station was conducted to evaluate within-lake spatial transfer. All observations from the same station were assigned exclusively to either training or testing within each fold. Feature standardization and model training used only the training stations, and all comparison models used identical station partitions.

##### Leave-One-Sampling-Event-Out Validation

To evaluate transfer to previously unseen sampling events and eliminate train–test sharing within the same field campaign, 17-fold leave-one-sampling-event-out (LOEO) validation was conducted. In each fold, all observations from one sampling event were held out for testing and the remaining 16 events were used for training. Standardization parameters were estimated from the training events only.

FDEGF and the Optical-only, No-gate, and Constant-gate structures were trained under 10 network initializations for each held-out event, and the median OOS prediction across initializations was used for evaluation. Predictions from all 17 held-out events were pooled to calculate overall $R^{2}$, RMSE, and MAE, while event-level MAE was used for paired structural comparisons. The station-grouped and event-grouped experiments therefore evaluate spatial and sampling-event transfer, respectively.

### 3.5. Retrieval-Performance Metrics

The coefficient of determination ($R^{2}$), root mean squared error (RMSE), and mean absolute error (MAE) were used to evaluate Chl-*a* retrieval performance:

(32) $$R^{2}=1-\frac{\sum_{i=1}^{n}{\left(y_{i}-{\hat{y}}_{i}\right)}^{2}}{\sum_{i=1}^{n}{\left(y_{i}-\bar{y}\right)}^{2}},$$

(33) $$RMSE=\sqrt{\frac{1}{n}\sum_{i=1}^{n}{\left(y_{i}-{\hat{y}}_{i}\right)}^{2}},$$

and

(34) $$MAE=\frac{1}{n}\sum_{i=1}^{n}\left|y_{i}-{\hat{y}}_{i}\right|.$$

Here, $y_{i}$ and ${\hat{y}}_{i}$ denote the measured and retrieved Chl-*a* concentrations, respectively, $\bar{y}$ is the mean measured concentration of the evaluated samples, and *n* is the number of evaluated observations. RMSE and MAE were calculated on the original concentration scale ($\mu g\;L^{-1}$). A negative $R^{2}$ indicates performance below a constant predictor equal to the mean measured concentration of the evaluated samples.

For the 31 paired random partitions, model differences were summarized using the median and interquartile range of paired metric differences together with better–worse counts. Because these partitions reuse the same observations, the across-partition summaries were treated as resampling-based stability descriptors rather than independent experimental replicates.

### 3.6. Use of Generative Artificial Intelligence in Manuscript Preparation

Generative artificial intelligence was used during manuscript preparation to assist with English translation and language editing, including improvements in clarity and organization. All scientific content, methodological descriptions, numerical results, and interpretations were reviewed and verified by the authors, who take full responsibility for the final manuscript.

## 4. Results

### 4.1. Optical Retrieval Across Wind-Speed Groups

To examine whether the optical Chl-*a* retrieval relationship varied under different wind-speed conditions, the samples were divided into low-, medium-, and high-wind-speed groups. The three groups contained 108, 91, and 88 samples, respectively, and each group covered all 20 sampling stations. Their median wind speeds were 1.672, 2.611, and $3.807\;m\;s^{-1}$, with corresponding ranges of 1.255–2.030, 2.312–3.393, and 3.489–$4.531\;m\;s^{-1}$.

**Figure 5:**
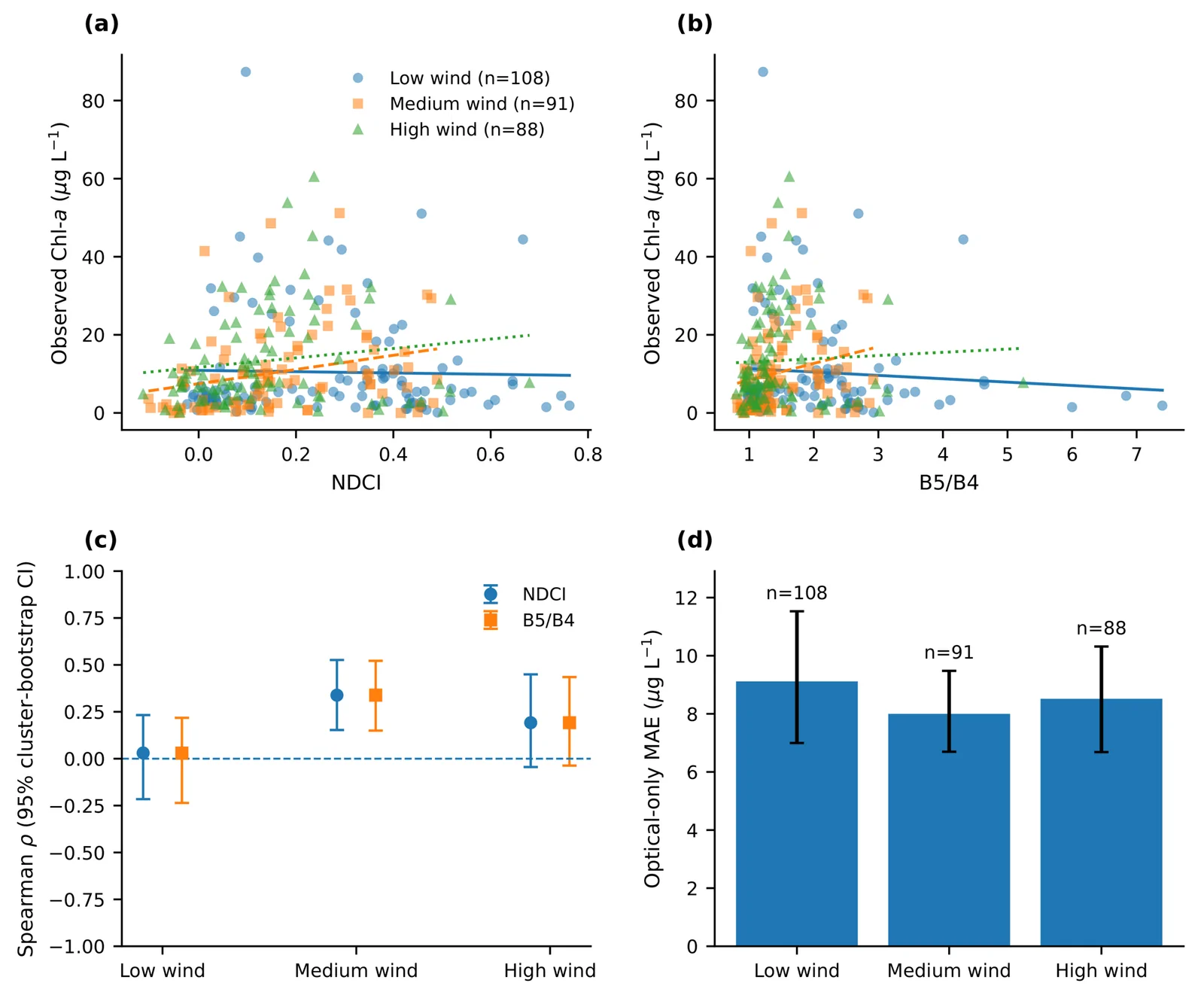
Optical feature–Chl-*a* relationships and optical retrieval errors across wind-speed groups. (**a**) NDCI and measured Chl-*a*; (**b**) B5/B4 and measured Chl-*a*. In panels (**a**) and (**b**), blue circles, orange squares, and green triangles represent low-, medium-, and high-wind-speed groups, respectively. The corresponding solid, dashed, and dotted lines indicate group-specific linear fits. (**c**) Spearman correlations with station-cluster bootstrap 95% confidence intervals; the horizontal dashed line indicates zero correlation. (**d**) MAE of the Optical-only model with station-cluster bootstrap 95% confidence intervals.

Figure 5a–c show that the dispersion, fitted relationships, and correlation strengths of both NDCI and B5/B4 differed among the three wind-speed groups. The bootstrap intervals also differed in width, indicating variable uncertainty in the estimated optical feature–Chl-*a* relationships. Similar optical-feature values could correspond to different Chl-*a* distributions across the groups, and this pattern was observed for both the normalized-difference and reflectance-ratio representations.

The MAE values of the Optical-only model were 9.116, 7.998, and $8.517\;\mu g\;L^{-1}$ in the low-, medium-, and high-wind-speed groups, respectively. The error decreased from the low- to the medium-wind-speed group and then increased in the high-wind-speed group, rather than following a monotonic wind-speed gradient. Together, the feature–concentration relationships and retrieval errors indicate that the performance of a fixed optical mapping was not uniform across the wind-speed groups. These results provide the observational context for the subsequent multimodal fusion analyses.

### 4.2. Overall Retrieval Performance and Error Characteristics

#### 4.2.1. Overall Retrieval Performance

The retrieval performance of FDEGF and the comparison models was evaluated using the same 31 paired random 80:20 train–test partitions with random states 0–29 and 42. Because model performance varied across partitions, test-set results were summarized using the median and interquartile range (IQR). The number of partitions with $R^{2}>0$ was additionally reported to describe how consistently each model performed above the corresponding test-set mean baseline. These repeated partitions were interpreted as resampling-based stability summaries rather than independent experimental replicates.

Table 1 shows that the nonlinear models generally outperformed Ridge, whose median $R^{2}$ was close to zero and which achieved positive $R^{2}$ in only 16 of the 31 partitions. This contrast indicates that a simple linear mapping was insufficient to represent the relationships between the multisource observations and Chl-*a* in the present dataset.

RF achieved the highest median $R^{2}$ and the lowest median RMSE, whereas SVR obtained the lowest median MAE and positive $R^{2}$ in all 31 partitions. XGBoost also maintained positive median $R^{2}$ and comparatively stable absolute-error performance. The relative performance of the models therefore depended on the evaluation metric, with $R^{2}$/RMSE and MAE emphasizing different aspects of retrieval error.

FDEGF achieved a median $R^{2}$ of 0.130 and positive $R^{2}$ in 23 of the 31 partitions. Its median $R^{2}$ was slightly higher than that of MLP, whereas its median RMSE and MAE were higher. One unfavorable partition produced $R^{2}=-3.285$ and RMSE $=23.399\;\mu g\;L^{-1}$, producing a long lower tail in the $R^{2}$ distribution and illustrating sensitivity to sample composition. The median and IQR were therefore used to describe typical performance across the repeated partitions.

FDEGF did not uniformly outperform the strongest conventional nonlinear baselines in overall retrieval accuracy. Its contribution was therefore evaluated further through paired structural controls and diagnostic experiments designed to determine whether the function-decoupled, event-conditioned fusion structure provided consistent structural benefits across the repeated partitions beyond unrestricted multimodal fusion.

#### 4.2.2. Out-of-Sample Retrieval-Error Characteristics

For each of the 287 unique field samples, retrievals obtained only from partitions in which that sample belonged to the test set were aggregated using their median. Each field record therefore contributed once to the sample-level OOS diagnostic. The OOS residual was defined as

(35) $$e_{i}^{\mathrm{OOS}}={\tilde{y}}_{i}^{\mathrm{OOS}}-y_{i},$$

where ${\tilde{y}}_{i}^{\mathrm{OOS}}$ denotes the median aggregated OOS retrieval for sample *i*. Positive and negative residuals indicate overestimation and underestimation, respectively.

**Figure 6:**
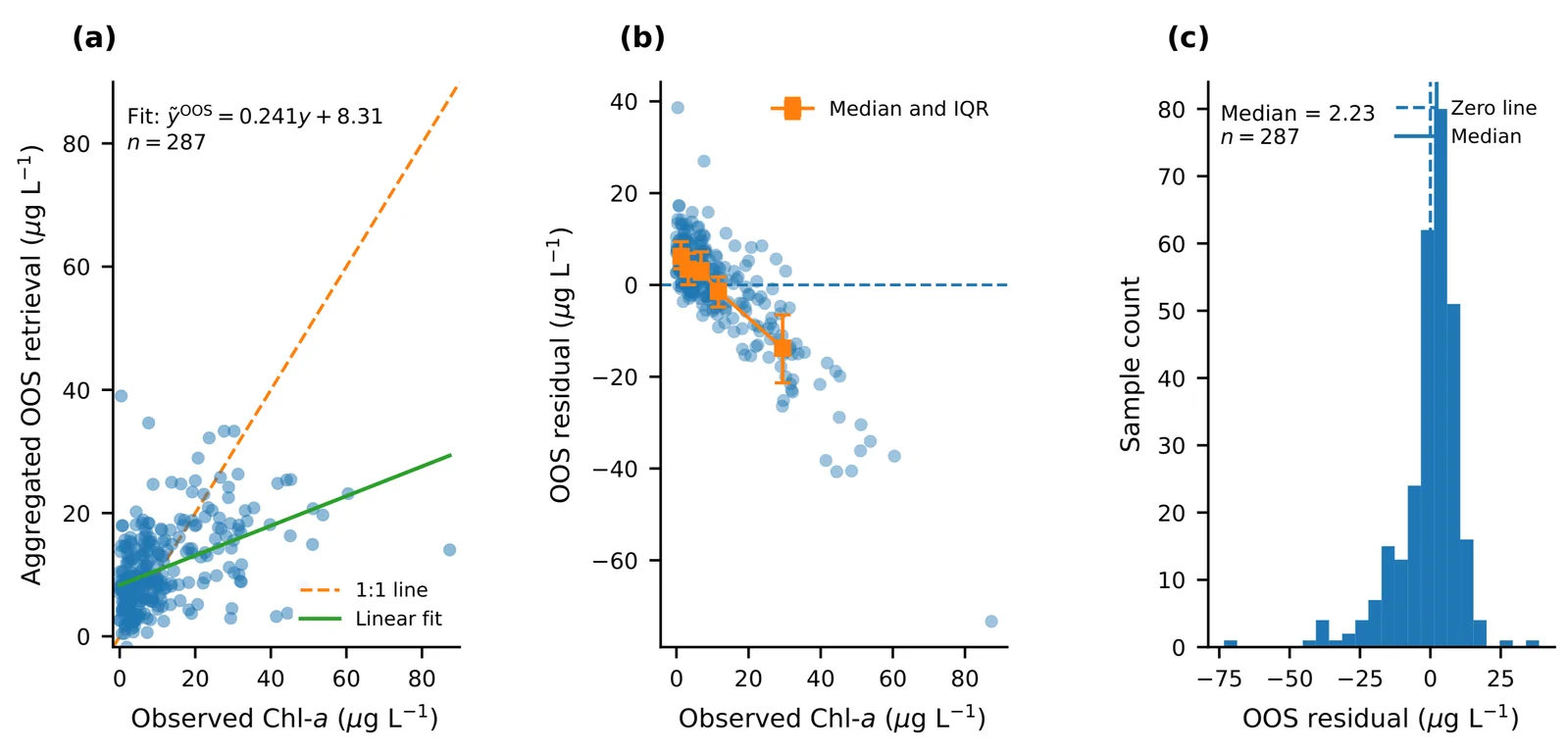
Aggregated OOS retrieval results and error characteristics for 287 unique samples. (**a**) Measured values versus median aggregated OOS retrievals; (**b**) residuals across measured concentrations, with five equal-frequency groups; and (**c**) residual-frequency distribution.

Figure 6a–c show that the aggregated OOS retrievals generally increased with measured Chl-*a*, indicating that FDEGF captured the overall direction of concentration variation. However, the fitted relationship,

$${\tilde{y}}^{\mathrm{OOS}}=0.241y+8.31,$$

had a slope substantially below unity. Low- and intermediate-concentration samples were generally closer to the 1:1 line, whereas low concentrations tended to be overestimated and high concentrations were increasingly underestimated.

The residual medians of the five equal-frequency concentration groups shifted from positive to negative values as measured Chl-*a* increased. The overall residual median was $2.23\;\mu g\;L^{-1}$, with both positive and negative tails. Together with the slope below unity, this pattern indicates compression of the retrieved concentration range toward the central part of the observed distribution rather than a uniform additive bias.

Errors were more dispersed at high concentrations. Only 27 samples in the final dataset had Chl-*a* concentrations of at least $30\;\mu g\;L^{-1}$. Because these high-concentration observations were relatively scarce, their allocation between training and test sets could strongly affect RMSE and $R^{2}$ across random partitions. High-concentration underestimation and the limited representation of the upper concentration range therefore remain important sources of retrieval uncertainty.

### 4.3. Structural Ablation Results

**Figure 7:**
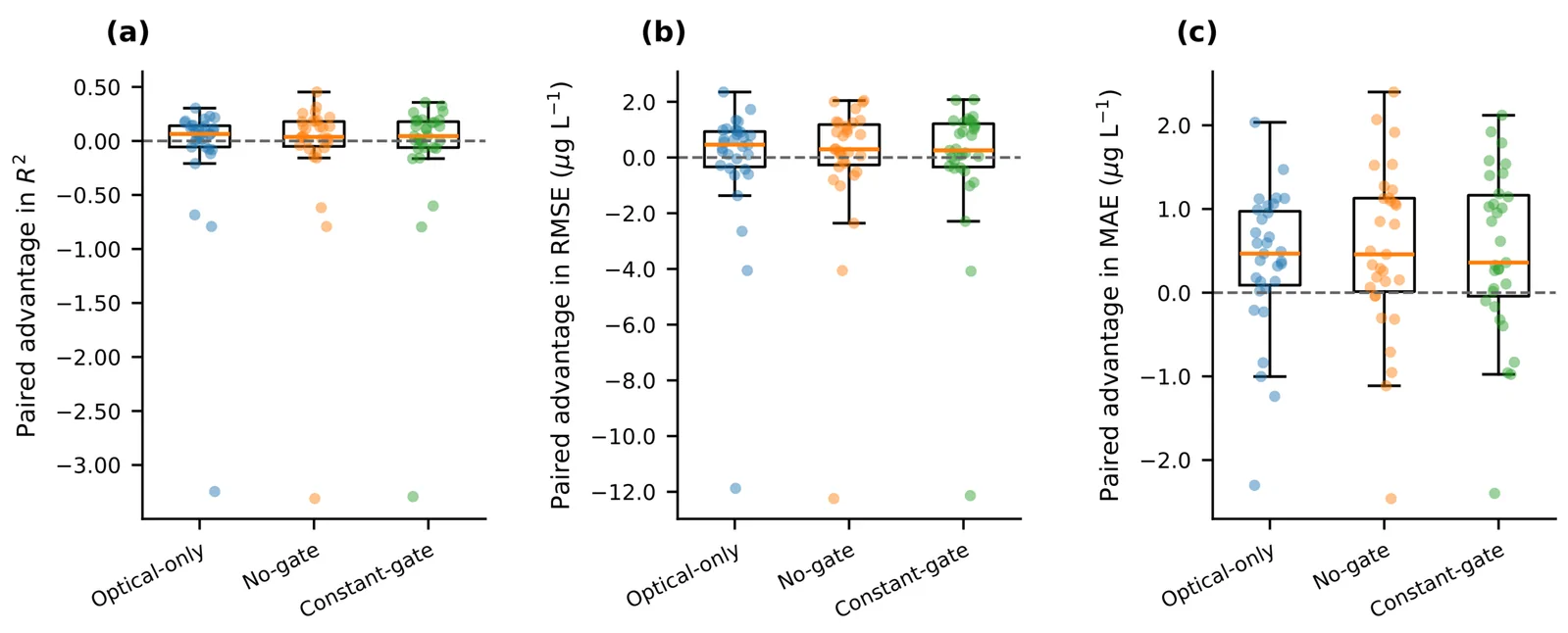
Paired performance advantages of FDEGF relative to the structural controls: (**a**) $R^{2}$; (**b**) RMSE; and (**c**) MAE. Positive values indicate better performance by FDEGF. The distributions summarize the 31 paired random partitions, and the horizontal reference line denotes zero paired advantage.

To evaluate whether the function-decoupled gated structure provided benefits beyond simpler multimodal fusion, FDEGF was compared with the Optical-only, No-gate, and Constant-gate structures under the same 31 paired random train–test partitions. As shown in Figure 7, paired advantages were defined such that positive values indicate better performance by FDEGF.

Relative to the Optical-only structure, FDEGF achieved median paired advantages of 0.064 in $R^{2}$, $0.462\;\mu g\;L^{-1}$ in RMSE, and $0.467\;\mu g\;L^{-1}$ in MAE. The $R^{2}$ advantage had an IQR of [−0.056,0.141], whereas the MAE advantage was more concentrated at $\left[0.089,0.973\right]\;\mu g\;L^{-1}$. FDEGF improved $R^{2}$ and RMSE in 21 of the 31 partitions and reduced MAE in 25 partitions.

Compared with the No-gate structure, the median paired advantages were 0.038 in $R^{2}$, $0.293\;\mu g\;L^{-1}$ in RMSE, and $0.458\;\mu g\;L^{-1}$ in MAE. FDEGF produced lower MAE in 23 of the 31 partitions. This comparison indicates that introducing the SAR auxiliary pathway without gating did not provide the same typical absolute-error reduction as the gated fusion structure.

Relative to the Constant-gate structure, FDEGF achieved median paired advantages of 0.044 in $R^{2}$, $0.258\;\mu g\;L^{-1}$ in RMSE, and $0.360\;\mu g\;L^{-1}$ in MAE, with lower MAE in 23 of the 31 partitions. Thus, event-conditioned gating provided an additional typical absolute-error advantage beyond using a single learnable scaling coefficient shared across all observations.

Across the three structural comparisons, the median paired advantages were positive for all three metrics, although the $R^{2}$ and RMSE differences varied more strongly among partitions. The clearest and most consistent structural benefit was observed for MAE, indicating that the principal gain of FDEGF under the repeated random-partition setting was improved control of typical absolute retrieval errors rather than uniform improvement in explained variance or large-error behavior.

#### 4.3.1. Temporal-Matching Sensitivity

The full-data structural comparison above was used as the reference condition, and the analysis was repeated after progressively restricting the satellite–ground temporal offsets. Table 2 summarizes the four restricted subsets.

The median MAE advantage of FDEGF remained positive relative to all three structural controls under each restricted matching criterion. In particular, when both Sentinel-1 and Sentinel-2 observations were restricted to within 5 d of field sampling, FDEGF achieved a median $R^{2}$ of 0.322 and reduced MAE relative to the Optical-only, No-gate, and Constant-gate structures by median values of 0.731, 0.901, and $0.997\;\mu g\;L^{-1}$, respectively. Lower MAE was obtained in 26 of the 31 paired partitions for all three comparisons.

Under the stricter joint 3-d criterion, only 110 samples from seven sampling events remained. The median paired MAE advantages remained positive, but the absolute retrieval performance and across-partition stability deteriorated. The persistence of the paired advantages after excluding the longer temporal offsets indicates that the structural differences observed in the full dataset were not solely attributable to the 12-d or 6-d Sentinel-1 matches. However, differences in absolute performance among the temporal subsets should not be interpreted as causal effects of temporal matching because the number of sampling events and the Chl-*a* distribution also changed with the filtering criteria.

#### 4.3.2. Recorded-Zero Sensitivity

Excluding the four observations recorded as $0.0\;\mu g\;L^{-1}$ produced only small median changes in FDEGF performance when evaluated on the common nonzero test samples: +0.0007 in $R^{2}$, $-0.006\;\mu g\;L^{-1}$ in RMSE, and $+0.005\;\mu g\;L^{-1}$ in MAE. The paired comparisons with the Optical-only, No-gate, and Constant-gate structures were also preserved. The aggregate structural conclusions were therefore not driven by the four recorded-zero observations.

### 4.4. Structural Diagnostics of Multimodal Fusion

#### 4.4.1. Sample-Level Benefits and Initialization Sensitivity

The unique-sample OOS analysis was first used to examine whether the structural benefit of FDEGF was distributed across individual observations. Relative to the independently trained Optical-only structure, the median sample-level absolute-error benefit was $1.479\;\mu g\;L^{-1}$, with lower absolute error for 63.4% of the 287 samples. Relative to the No-gate structure, the corresponding median benefit was $1.715\;\mu g\;L^{-1}$, with 64.5% of samples benefiting. Thus, the absolute-error advantage observed in the paired structural comparison was distributed across a substantial proportion of the dataset rather than being driven by a small number of observations.

Because the three pathways were optimized jointly using only the final retrieval loss, the numerical decomposition of the fitted model was further examined under different network initializations. FDEGF was retrained on the same original single random partition using 20 independent initializations while all other training settings were held fixed.

**Figure 8:**
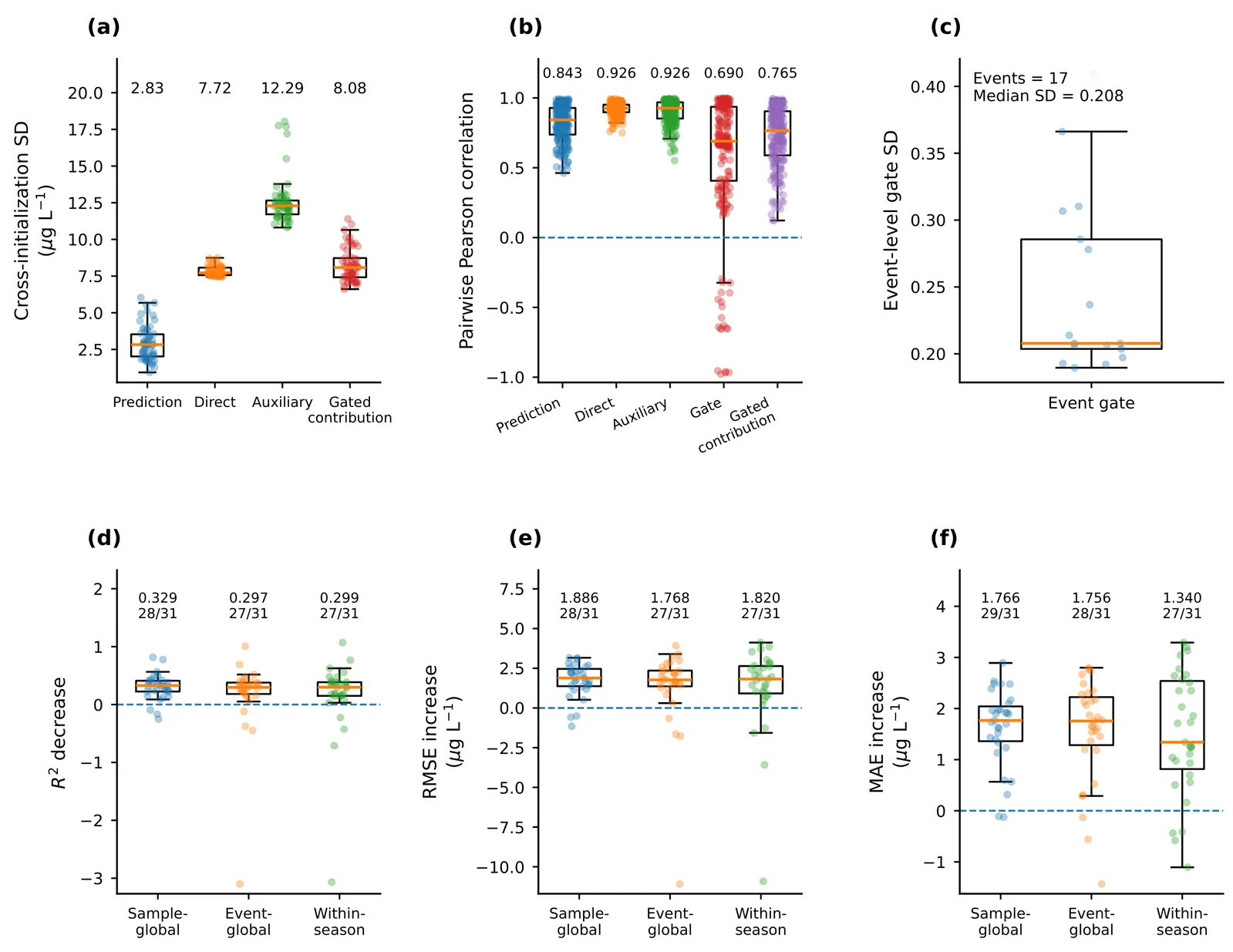
Structural diagnostics of FDEGF under network re-initialization and gate mismatch. (**a**) Sample-wise cross-initialization standard deviations of the final prediction, direct output, auxiliary output, and gated auxiliary contribution; (**b**) pairwise Pearson correlations of the final prediction and internal pathway outputs across 20 network initializations; (**c**) event-level variability of the learned gate across initializations; and (**d**–**f**) retrieval-performance degradation under sample-global, event-global, and within-season event-level gate mismatch. Numbers above panels (**a**,**b**) denote the corresponding median values. For panels (**d**–**f**), each point represents the median degradation over repeated gate perturbations within one of the 31 paired random partitions. Positive values indicate performance degradation after gate mismatch.

Figure 8a–c show that the final retrieval was less variable in absolute magnitude across initializations than the internal direct, auxiliary, and gated auxiliary contribution outputs. The direct and auxiliary outputs nevertheless retained high cross-initialization pattern agreement, with both showing a median pairwise Pearson correlation of 0.926. The corresponding median correlations were 0.843 for the final prediction, 0.690 for the gate, and 0.765 for the gated auxiliary contribution.

The event-level gate also showed appreciable variation across initializations. These results indicate that the architecture produced partially reproducible pathway patterns, but the numerical values assigned to the individual branches were not uniquely identified by the final MSE objective. The direct, auxiliary, and gating outputs should therefore be interpreted as architecture-dependent computational components rather than uniquely separable physical quantities.

#### 4.4.2. Event-Constrained Gate-Mismatch Analysis

Gate mismatch was re-evaluated using perturbations consistent with the event-level structure of the meteorological inputs. The original sample-global reassignment was retained as a reference, while complete event-level gate blocks were additionally reassigned either across all sampling events or only among events within the same season.

As shown in Figure 8d–f, all three perturbation schemes degraded retrieval performance in most random partitions. Under event-global reassignment, the median $R^{2}$ decrease was 0.297, while RMSE and MAE increased by median values of 1.768 and $1.756\;\mu g\;L^{-1}$, respectively. Restricting gate reassignment to events within the same season produced a similar median $R^{2}$ decrease of 0.299 and median RMSE and MAE increases of 1.820 and $1.340\;\mu g\;L^{-1}$, respectively.

The persistence of performance degradation after preserving complete sampling-event blocks, and even coarse seasonal organization, indicates that the fitted model depended on the correspondence between the event-conditioned gate and the SAR auxiliary pathway. This intervention does not establish a unique physical regulation mechanism, but it provides intervention-based evidence that the learned gate–auxiliary correspondence contributes to the operation of the fitted fusion structure.

The initialization and mismatch results are complementary rather than contradictory. Within a fitted parameterization, disrupting the learned gate–auxiliary correspondence degraded retrieval performance, whereas different network initializations could realize this dependence through different internal numerical decompositions.

#### 4.4.3. Input-Pathway Permutation Analysis

The dependence of FDEGF on the three input pathways was further examined through grouped permutation of the optical, meteorological, and SAR feature groups. Global, within-year, and within-season permutations were considered, with all variables within the same feature group permuted jointly.

Optical permutation produced median $R^{2}$ decreases of 0.129, 0.115, and 0.135 under the global, within-year, and within-season schemes, respectively, with retrieval performance deteriorating in most random partitions. Meteorological permutation produced larger median $R^{2}$ decreases of 0.289, 0.207, and 0.238 under the corresponding schemes. Under global permutation, RMSE and MAE increased by median values of 1.858 and $1.702\;\mu g\;L^{-1}$, respectively. SAR permutation produced weaker changes, with a median $R^{2}$ decrease of 0.044 under global permutation and values below 0.02 under the restricted schemes.

These differences indicate that the fitted model depends differently on the three architecture-assigned information pathways. The permutation magnitude should not be interpreted as a direct ranking of sensing-source importance, because the three feature groups enter different computational pathways and therefore perturb different parts of the model. In particular, the meteorological variables condition the gating pathway rather than providing a direct Chl-*a* retrieval signal.

Because the meteorological inputs are shared by stations within the same sampling event, the within-year and within-season permutation results are treated here as pathway-dependence diagnostics rather than evidence of sample-specific meteorological regulation. Event-preserving correspondence was evaluated separately by the gate-mismatch analysis described above.

#### 4.4.4. Branch Behavior Across Wind-Speed Groups

**Figure 9:**
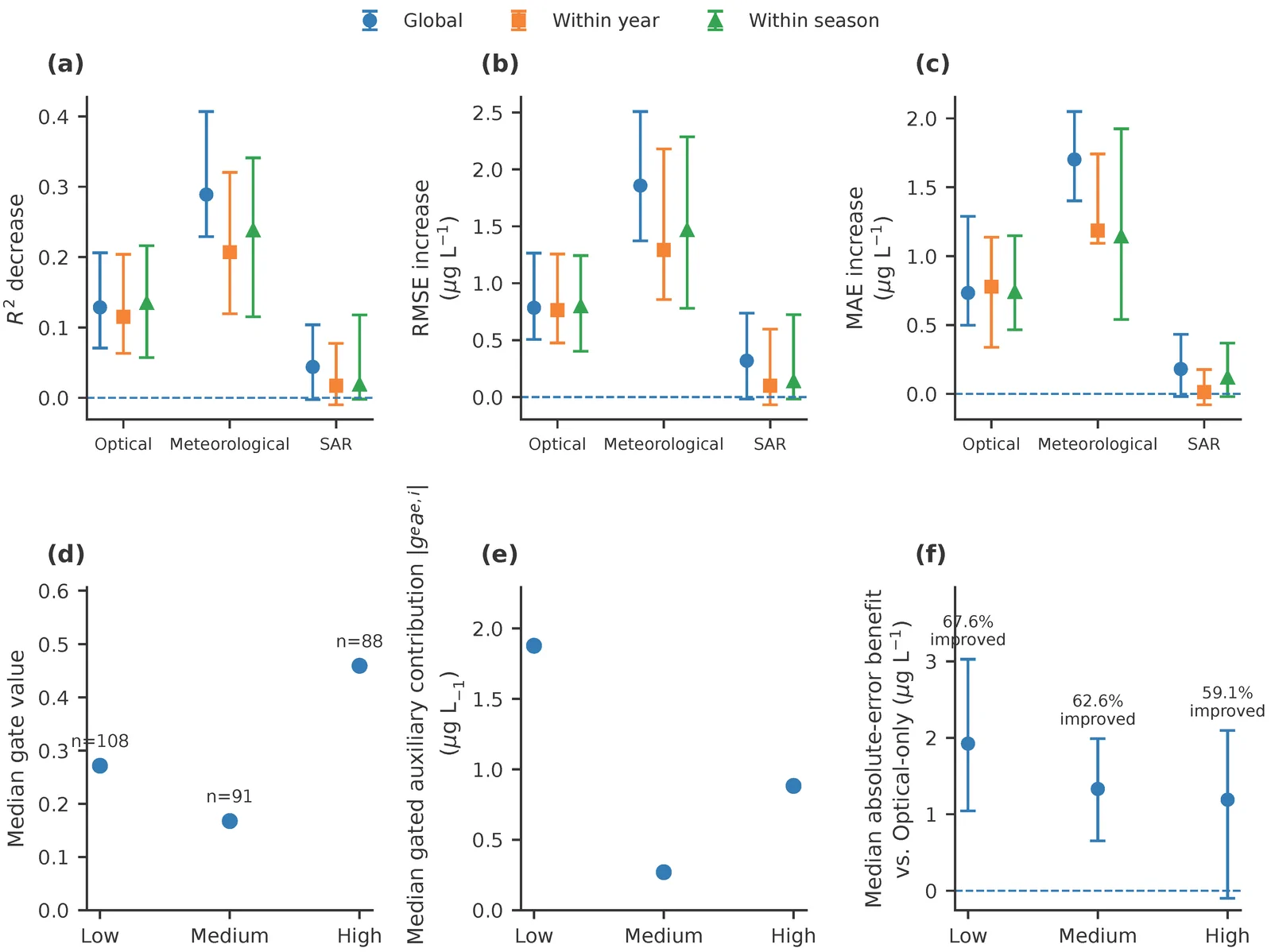
Input-pathway permutation and branch behavior across wind-speed groups. (**a**–**c**) Changes in $R^{2}$, RMSE, and MAE after optical, meteorological, and SAR feature-group permutation under global, within-year, and within-season schemes; (**d**) median gate values; (**e**) median absolute gated auxiliary contributions; and (**f**) median sample-level absolute-error benefits relative to the Optical-only structure across wind-speed groups. The grouped pathway responses are interpreted as diagnostics of model dependence rather than as a ranking of physical variable importance.

Figure 9d–f provide a descriptive comparison of the learned branch behavior across the low-, medium-, and high-wind-speed groups. The median gate values were 0.271, 0.167, and 0.459, respectively, whereas the corresponding median absolute gated auxiliary contributions were 1.877, 0.270, and $0.882\;\mu g\;L^{-1}$. Thus, larger gate values did not necessarily correspond to larger effective auxiliary contributions.

The median absolute-error benefits relative to the independently trained Optical-only structure were 1.924, 1.331, and $1.191\;\mu g\;L^{-1}$ for the low-, medium-, and high-wind-speed groups, respectively, with 67.6%, 62.6%, and 59.1% of samples showing lower absolute error.

These non-monotonic group patterns show that the effective auxiliary contribution depends jointly on the event-conditioned gate and the station-dependent SAR auxiliary output. Because the gate is generated from both wind speed and air temperature, and because its numerical value is initialization-sensitive, the groupwise gate values are interpreted as descriptive model outputs rather than as direct physical responses to wind speed.

#### 4.4.5. SAR Geometry and Association Diagnostics

Additional diagnostics were conducted to clarify the interpretation of the SAR auxiliary pathway. All 287 samples were acquired under the same Sentinel-1A ascending-orbit and relative-orbit configuration, but the local incidence angle still varied from approximately 35.064° to 36.421°. The association between incidence angle and VV backscatter was appreciable (sample-level Pearson r=0.423), indicating that residual within-lake viewing geometry remained relevant despite the controlled acquisition configuration.

In contrast, no stable pairwise association was detected between VV/VH backscatter and near-contemporaneous wind conditions or between VV/VH and independent Optical-only OOS retrieval residuals. Similar conclusions were obtained after accounting for incidence-angle variation. The SAR pathway is therefore interpreted as an architecture-dependent auxiliary information source rather than as a directly identified physical residual-correction mechanism.

### 4.5. Grouped Transfer Validation

#### 4.5.1. Unseen-Station Validation

Five-fold cross-validation grouped by sampling station was used to evaluate retrieval at locations excluded from model training. All observations from the same station were assigned exclusively to either the training or test subset within each fold, and data standardization and model fitting were performed using the training stations only. This design prevented observations collected at the same station on different dates from appearing in both training and testing.

FDEGF achieved a mean test-set $R^{2}$ of 0.235±0.154, compared with 0.245±0.201 for RF (Table 3). Thus, both models retained positive mean $R^{2}$ when complete sampling locations were excluded from model training. The interfold standard deviation was numerically smaller for FDEGF, although the five-fold design does not support a strong conclusion regarding differences in spatial stability.

Station-grouped validation imposes spatial separation between training and testing samples, but sampling events can still be represented on both sides of a fold. The result therefore supports partial transfer across sampling locations within the observed temporal domain rather than transfer to previously unseen sampling events.

#### 4.5.2. Leave-One-Sampling-Event-Out Validation

To evaluate transfer to previously unseen sampling events, 17-fold leave-one-sampling-event-out (LOEO) validation was conducted. In each fold, all observations from one complete sampling event were withheld for testing, while the remaining 16 events were used for model training. Predictions from the 17 held-out events were pooled for overall evaluation.

As shown in Table 4, performance decreased substantially under event-independent validation. The pooled $R^{2}$ values were 0.002 for Optical-only, −0.029 for No-gate, −0.023 for Constant-gate, and −0.144 for FDEGF. FDEGF yielded an RMSE of $13.420\;\mu g\;L^{-1}$ and an MAE of $9.849\;\mu g\;L^{-1}$.

For each of the three structural-control comparisons, FDEGF yielded a lower event-level MAE in 7 of the 17 held-out events, indicating that the structural advantage observed under the repeated random partitions did not persist consistently when an entire sampling event was unseen during training.

The two grouped evaluations therefore define different transfer boundaries. The unseen-station experiment indicates that relationships learned from the observed sampling period can retain retrieval capability at held-out locations, whereas the LOEO experiment shows that the present model does not yet provide robust transfer to previously unseen sampling events. Accordingly, the repeated random-partition results reported above are interpreted as within-domain retrieval and structural-behavior evaluations, rather than as evidence of event-independent generalization.

## 5. Discussion

### 5.1. Observation-Condition Heterogeneity and the Rationale for Role-Aware Fusion

Satellite-based Chl-*a* retrieval in shallow inland waters is affected not only by phytoplankton-related optical signals but also by suspended materials, bottom-related effects, and changes in water-surface and meteorological conditions. Wind-induced resuspension can alter suspended-material concentrations and water-leaving reflectance, while differences among optical water types can further modify the relationship between spectral observations and Chl-*a* [14,15,16,18,19]. These effects are particularly relevant to shallow lakes such as Lake Ulansuhai, where optical conditions are spatially and temporally heterogeneous [17].

The results in Figure 5 are consistent with this observation-condition heterogeneity. Both NDCI and B5/B4 showed different feature–Chl-*a* relationships across the three wind-speed-defined groups, and Optical-only retrieval errors also differed among groups. Importantly, MAE did not increase monotonically with wind speed. These differences should therefore not be interpreted as a simple causal relationship in which stronger wind necessarily produces poorer optical retrieval. Rather, they indicate that the suitability of a fixed optical mapping can vary across the observation conditions represented in the current dataset.

This heterogeneity provides the main motivation for the role-aware fusion strategy adopted in FDEGF. Optical observations contain the most direct spectral information related to Chl-*a*, whereas SAR observations provide complementary backscatter information related to surface-scattering conditions and the meteorological variables describe the event-level background under which the observations were acquired. Recent multimodal remote-sensing studies have similarly shown the value of modality-specific branches, gated fusion, and dynamic information weighting when heterogeneous observations contribute differently to a task [25,26,27,28].

FDEGF therefore does not treat the three modalities as interchangeable predictors. Instead, Sentinel-2 optical information forms the direct retrieval pathway, Sentinel-1 SAR information provides an auxiliary pathway, and the meteorological descriptors condition the participation of that pathway at the sampling-event level. The resulting architecture makes the assigned roles of heterogeneous information sources explicit and experimentally testable, rather than relying only on an implicit joint mapping of all input variables.

### 5.2. Overall Accuracy Versus Structural Benefit

The overall model comparison in Table 1 defines an important boundary for interpreting the contribution of FDEGF. RF achieved the highest median $R^{2}$ and the lowest median RMSE, while SVR achieved the lowest median MAE. FDEGF did not uniformly outperform these strong nonlinear baselines and was more sensitive to several unfavorable sample partitions. The present results therefore do not support describing FDEGF as a universally superior retrieval model on the basis of aggregate accuracy alone.

Rather, the proposed framework addresses a more specific question: whether heterogeneous sensing information can be assigned distinct computational roles and whether event-conditioned gating of the auxiliary pathway yields a consistent structural benefit across repeated partitions within the represented sampling-event domain. Conventional nonlinear regression models can effectively exploit the available predictors, and their strong performance confirms that they remain competitive approaches for Chl-*a* retrieval.

The ablation results in Figure 7 support this structural interpretation. Relative to the Optical-only, No-gate, and Constant-gate structures, FDEGF reduced median MAE by 0.467, 0.458, and $0.360\;\mu g\;L^{-1}$, respectively. Lower MAE was obtained in 25 of the 31 paired random partitions relative to Optical-only and in 23 of the 31 partitions relative to both No-gate and Constant-gate. The corresponding $R^{2}$ and RMSE advantages were positive in direction across many partitions but were more sensitive to sample composition.

The principal structural benefit should therefore be interpreted as a more consistent reduction in typical absolute retrieval error within the observed domain, rather than as a uniform improvement in explained variance or extreme-error behavior. This interpretation is also consistent with the OOS results in Figure 6, where the retrieved concentration range was compressed and a small number of high-concentration observations produced substantially larger residuals. These observations exerted stronger effects on RMSE and $R^{2}$ than on MAE.

The three structural controls further separate the roles of different design choices. Improvement over the Optical-only structure indicates that information beyond the direct optical pathway can contribute to retrieval. Across the repeated partitions, the advantage over the No-gate structure indicates that unrestricted addition of the auxiliary output was less consistently effective for typical absolute-error reduction than gated participation. Similarly, the advantage over the Constant-gate structure indicates that a single global scaling coefficient did not reproduce the same level of absolute-error reduction as event-conditioned gating across the repeated partitions.

Accordingly, the methodological contribution of FDEGF lies not in replacing all conventional nonlinear models, but in providing a functionally organized multimodal fusion architecture in which the participation of heterogeneous sensing information can be examined through controlled structural comparisons.

### 5.3. Structural Interpretation of Function-Decoupled Multimodal Fusion

The structural diagnostics provide complementary evidence for how FDEGF uses its architecture-assigned information pathways, while also defining an important boundary for interpreting the internal branch outputs. Under the fixed seed-42 train–test partition, the initialization-stability analysis showed that the final retrieval had lower cross-initialization amplitude variability than the internal direct, auxiliary, and gated-contribution outputs. The direct and auxiliary pathways nevertheless retained relatively consistent sample-wise patterns. Because all branches were jointly optimized through the final fused retrieval loss, these results indicate that the functional organization of the architecture is partly reproducible, whereas its numerical internal decomposition is not uniquely identified. The branch outputs should therefore be interpreted as architecture-dependent computational components rather than independently identified physical quantities.

Within this identifiability boundary, the gate-mismatch experiment provides intervention-based evidence that the correspondence between the meteorological gate and the auxiliary pathway matters to the fitted model. Reassigning complete event-level gate blocks among sampling events caused a median $R^{2}$ decrease of 0.297 and median RMSE and MAE increases of 1.768 and $1.756\;\mu g\;L^{-1}$, respectively. Similar degradation remained when event-level gates were reassigned only among sampling events within the same season, with a median $R^{2}$ decrease of 0.299 and RMSE and MAE increases of 1.820 and $1.340\;\mu g\;L^{-1}$. Thus, the mismatch effect cannot be attributed solely to disruption of within-event structure or coarse seasonal organization. Rather, it indicates that retrieval performance depends on maintaining the learned correspondence between the meteorological background of a sampling event and the SAR-derived auxiliary pathway.

The grouped permutation analysis provides a complementary view of this pathway dependence. Optical permutation consistently degraded retrieval, supporting the assigned role of optical information as the principal direct retrieval source. Meteorological permutation also produced substantial performance changes, including under within-year and within-season constraints. This response should not be interpreted as showing that wind speed and air temperature contain more direct Chl-*a* information than the optical observations. The meteorological variables enter the gating pathway rather than the direct retrieval pathway, and their permutation therefore perturbs a different computational function. Because the meteorological variables are shared by stations within the same sampling event, these restricted permutations should be interpreted as pathway-dependence diagnostics rather than evidence of within-event meteorological regulation. Their persistence shows that the observed dependence is not explained solely by broad year or season categories, whereas event-preserving correspondence is evaluated more directly by the gate-mismatch experiments described above.

SAR permutation produced weaker and less consistent changes, especially under the constrained schemes. This result is compatible with the auxiliary role assigned to SAR rather than implying that SAR information is unnecessary. Sentinel-1 observations are sensitive to surface-scattering characteristics and have been used to characterize inland-water surface conditions and complement optical observations [20,21,22,23]. However, the additional SAR diagnostics in the present study showed that backscatter, particularly VV, was also sensitive to the remaining within-lake incidence-angle variation, whereas no stable association was identified between VV/VH and near-contemporaneous wind conditions or independent Optical-only OOS residuals. The SAR pathway is therefore better interpreted as an architecture-dependent auxiliary information source than as a uniquely identified physical residual-correction mechanism.

The wind-group analysis further illustrates why the gate itself should not be assigned a direct physical meaning. The high-wind-speed group exhibited the largest median gate value, whereas the low-wind-speed group showed the largest median gated auxiliary contribution and the largest median absolute-error benefit relative to Optical-only. Gate magnitude, auxiliary contribution, and retrieval benefit therefore did not vary synchronously across the three groups. In addition, the initialization analysis showed that the numerical gate values were sensitive to model initialization. Because the gating pathway jointly uses wind speed and air temperature, these groupwise gate values are best regarded as descriptive outputs of a fitted parameterization rather than direct physical responses to wind speed.

Taken together, the diagnostic experiments support architecture-dependent functional differentiation within FDEGF. Optical information provides the direct retrieval pathway, event-level meteorological descriptors condition the participation of the auxiliary pathway, and SAR provides complementary information whose effective contribution remains station-dependent through the auxiliary output. Disrupting the learned correspondence among these components degrades retrieval performance, but this dependence does not imply a unique physical decomposition or a causal hierarchy among sensing sources. The value of the function-decoupled design is therefore that heterogeneous information flows are explicitly organized and can be examined through controlled structural interventions.

### 5.4. Error Structure, Transferability, and Limitations

The OOS analysis reveals an important limitation that should be considered together with the structural improvements described above. As shown in Figure 6, the aggregated OOS relationship exhibited substantial dynamic-range compression, with a fitted slope of 0.241. Lower Chl-*a* concentrations tended to be overestimated, whereas higher concentrations were increasingly underestimated. The resulting error pattern is therefore concentration-dependent rather than a uniform additive bias.

This behavior is closely related to the available concentration distribution. Only 27 of the 287 observations had Chl-*a* concentrations of at least $30\;\mu g\;L^{-1}$, and this limited representation contributed to the larger and more variable residuals in the upper concentration range. This helps explain why RMSE and $R^{2}$ varied more strongly among random partitions, whereas the structural benefit in MAE was comparatively consistent. FDEGF therefore improved typical absolute errors for many observations but did not eliminate the limited representation of the upper concentration range.

The grouped validation results further define the transferability boundary of the present framework. Under five-fold unseen-station validation, FDEGF achieved $R^{2}=0.235\pm 0.154$, indicating that relationships learned from the observed sampling period retained retrieval capability at locations excluded from model fitting. This result should, however, be distinguished from event-independent transfer because sampling events can still be represented in both the training and test subsets of a station-grouped split.

The LOEO experiment provides a stricter test of this distinction. When one complete sampling event was excluded from training in each fold, FDEGF obtained a pooled $R^{2}$ of −0.144 and did not retain a consistent advantage over the three structural controls; for each comparison, lower event-level MAE was obtained in only 7 of the 17 held-out events. The repeated random partitions should therefore be interpreted primarily as evaluations of within-domain retrieval and structural behavior under sampling events already represented in the dataset, rather than as evidence of robust transfer to previously unseen events. The current framework has not yet solved event-domain generalization.

Temporal matching introduces a related source of uncertainty. The sensitivity analysis showed that the paired structural advantage in MAE remained observable after progressively restricting the satellite–field matchup windows, including when both Sentinel-1 and Sentinel-2 observations were limited to within 5 d of field sampling. Under the strict joint 3-d criterion, however, only 110 samples from seven sampling events remained and absolute retrieval performance became unstable. Differences among these temporal subsets cannot be attributed solely to temporal mismatch because stricter filtering also changed the retained sampling events and the Chl-*a* distribution. Denser field and satellite observations would allow temporal-coincidence effects to be evaluated with less confounding from sample-composition changes.

Several data and observation limitations also constrain the physical interpretation of the learned pathways. Sentinel-2 L2A products processed with Sen2Cor were used consistently throughout the study, but ACOLITE or another aquatic atmospheric-correction processor was not independently tested. Because concurrent in situ water-surface reflectance was unavailable, the present study cannot determine which atmospheric-correction approach is most accurate for Lake Ulansuhai. Residual atmospheric and adjacency-related effects may therefore remain in B4, B5, and their derived optical indices, and the interpretation of the direct optical pathway should be understood within the adopted L2A processing chain.

The SAR and meteorological inputs are also compact descriptors rather than complete representations of shallow-lake physical processes. As discussed above, SAR backscatter remains sensitive to acquisition geometry, while the daily mean wind speed and air temperature provide only coarse event-level meteorological conditioning. The present framework does not explicitly model wind duration, fetch, wave characteristics, sediment shear stress, bathymetric variation, bottom reflectance, or vegetation-mediated hydrodynamics. Wind-driven resuspension, bottom influence, and surface roughness should therefore be regarded as plausible sources of observation variability rather than processes explicitly resolved by FDEGF.

A further data limitation concerns four observations recorded as $0.0\;\mu g\;L^{-1}$. The original laboratory records and sample-specific detection or quantification limits were unavailable, so these records cannot be retrospectively classified as true zeros or below-detection-limit measurements. Excluding the four records produced only negligible median changes in the common-test evaluation, indicating that the main retrieval and structural conclusions were not driven by these values.

Finally, the framework was developed and evaluated using Lake Ulansuhai observations from 2017–2019. The unseen-station results support spatial transfer within the represented lake and observation period, whereas the LOEO results show that transfer to new sampling events remains substantially more difficult. Independent cross-lake datasets, longer observation periods, and broader optical and meteorological regimes are therefore required to determine whether the same functionally organized fusion strategy remains effective beyond the domain represented here.

Overall, the present evidence supports FDEGF as a role-aware and experimentally testable multimodal fusion framework within the represented observation domain. Its principal limitations concern high-concentration representation, event-domain transfer, temporal coincidence, optical preprocessing uncertainty, and the restricted physical information contained in the current SAR and meteorological descriptors. Accordingly, the strongest conclusion supported by the current evidence is limited to the represented observation domain: explicitly organizing direct, auxiliary, and event-conditioned information pathways provides a useful and experimentally testable basis for heterogeneous multimodal fusion. Generalization of the fitted relationships to previously unseen sampling events, longer temporal ranges, or other lakes remains to be established.

## 6. Conclusions

This study investigated multimodal Chl-*a* retrieval in Lake Ulansuhai, a cold–arid shallow lake with heterogeneous optical and observation conditions. A satellite–ground matched dataset containing 287 valid observations from 2017–2019 was constructed by integrating field-measured Chl-*a*, Sentinel-2 optical observations, Sentinel-1 SAR observations, and ERA5 meteorological descriptors. Based on this dataset, a function-decoupled environment-gated multimodal fusion framework, FDEGF, was developed in which optical information provides the direct retrieval pathway, SAR information provides an auxiliary pathway, and meteorological descriptors condition auxiliary participation at the sampling-event level.

The wind-speed-group analysis showed that the relationships between optical features and Chl-*a*, together with Optical-only retrieval errors, varied across observation conditions without following a simple monotonic wind-speed gradient. This heterogeneity supports the motivation for assigning different computational roles to heterogeneous information sources rather than treating all modalities as interchangeable predictors.

Across 31 paired random train–test partitions, the structural benefit of FDEGF was expressed most consistently in MAE. Relative to the Optical-only, No-gate, and Constant-gate structures, the median MAE reductions were 0.467, 0.458, and $0.360\;\mu g\;L^{-1}$, respectively. FDEGF achieved lower MAE in 25 of the 31 partitions relative to Optical-only and in 23 of the 31 partitions relative to both No-gate and Constant-gate. The corresponding $R^{2}$ and RMSE improvements were more sensitive to sample composition, and conventional nonlinear models remained competitive in aggregate retrieval accuracy. Sensitivity analyses further showed that the paired MAE advantage remained observable under stricter satellite–field temporal-matching criteria, although differences in absolute performance among temporal subsets were also affected by changes in event and target-distribution composition.

The structural diagnostics further clarified what can and cannot be inferred from the learned architecture. Event-preserving gate-mismatch experiments, including reassignment only among sampling events from the same season, continued to degrade retrieval performance, indicating that the fitted model depends on the correspondence between event-level meteorological gating and the SAR auxiliary pathway. At the same time, initialization analysis showed that the numerical direct, auxiliary, gate, and gated-contribution outputs were not uniquely identified across network initializations. Grouped permutation and SAR diagnostics similarly supported differentiated architecture-dependent pathway use, but not a unique physical decomposition. Accordingly, the learned branches should be interpreted as architecture-defined computational components rather than independently identified physical quantities.

The principal remaining limitations concern both error structure and transferability. Aggregated OOS retrievals exhibited dynamic-range compression, with increasing underestimation at high Chl-*a* concentrations where observations were relatively sparse. Under five-fold unseen-station validation, FDEGF achieved $R^{2}=0.235\pm 0.154$, indicating partial spatial transfer within the represented lake and observation period. In contrast, under 17-fold leave-one-sampling-event-out validation, FDEGF yielded a pooled $R^{2}$ of −0.144 and achieved lower event-level MAE than each structural control in only 7 of the 17 held-out events. The repeated random-partition results should therefore be interpreted as evidence of retrieval behavior and structural benefit within the represented sampling-event domain rather than as evidence of robust transfer to previously unseen events.

Overall, the contribution of FDEGF lies in providing a functionally organized and experimentally testable multimodal fusion framework rather than in uniformly outperforming conventional nonlinear models. The current evidence supports the use of explicit direct, auxiliary, and event-conditioned pathways for examining heterogeneous multimodal information within the represented observation domain. Future work should expand temporal and high-concentration coverage, improve optical preprocessing and SAR and meteorological characterization, reduce satellite–ground temporal discrepancies, and evaluate whether the same functional organization can generalize to previously unseen sampling events and independent lakes. 

## Figures and Tables

**Table 1:** Performance of different models across 31 paired repeated random partitions. Values are reported as median [interquartile range]. Bold values identify the best median result for each of $R^{2}$, RMSE, and MAE.

Model	$R^{2}$	RMSE ($\mu g\;L^{-1}$)	MAE ($\mu g\;L^{-1}$)	Partitions with $R^{2}>0$
Ridge	0.003 [−0.031, 0.029]	12.415 [10.962, 13.873]	9.184 [8.609, 9.888]	16/31
SVR	0.196 [0.132, 0.256]	11.078 [9.152, 13.169]	**7.310 [6.034, 7.628]**	31/31
RF	**0.254 [0.155, 0.330]**	**10.478 [9.269, 12.416]**	7.440 [6.797, 8.211]	30/31
XGBoost	0.183 [0.047, 0.243]	11.271 [10.162, 12.535]	7.792 [7.238, 8.289]	25/31
MLP	0.118 [−0.002, 0.264]	11.081 [9.521, 13.172]	7.914 [7.099, 8.733]	23/31
FDEGF	0.130 [−0.016, 0.239]	11.986 [10.216, 13.542]	8.249 [7.597, 9.174]	23/31

**Table 2:** Temporal-matching sensitivity of the FDEGF structural advantage. Positive ΔMAE denotes lower MAE for FDEGF. Values in parentheses indicate the number of paired partitions, out of 31, in which FDEGF achieved lower MAE than the corresponding structural control.

Matching Criterion	Samples	Events	FDEGF Median $R^{2}$	ΔMAE vs. Optical-Only	ΔMAE vs. No-Gate	ΔMAE vs. Constant-Gate
Exclude 12-d S1	271	16	0.044	0.385 (19/31)	0.594 (24/31)	0.609 (24/31)
S1 ≤5 d	252	15	0.143	0.318 (22/31)	0.450 (21/31)	0.386 (23/31)
S1/S2 ≤5 d	166	10	0.322	0.731 (26/31)	0.901 (26/31)	0.997 (26/31)
S1/S2 ≤3 d	110	7	−0.110	0.373 (20/31)	0.685 (19/31)	0.738 (19/31)

**Table 3:** Performance under five-fold unseen-station validation. Values are reported as mean ± standard deviation of test-set $R^{2}$.

Model	Folds	Test-Set $R^{2}$
FDEGF	5	0.235±0.154
RF	5	0.245±0.201

**Table 4:** Performance under leave-one-sampling-event-out validation.

Model	$R^{2}$	RMSE (μg L^−1^)	MAE (μg L^−1^)
Optical-only	0.002	12.532	9.165
No-gate	−0.029	12.729	9.380
Constant-gate	−0.023	12.692	9.339
FDEGF	−0.144	13.420	9.849

## Data Availability

The data supporting the findings of this study are available from the corresponding author upon reasonable request. The original in situ Chl-*a* concentration data are not publicly available because they were collected by the research team and are subject to the internal data-management and sharing requirements of the project group.

## Abbreviations

The following abbreviations are used in this manuscript:

Chl-*a*	Chlorophyll-*a*
FDEGF	Function-decoupled environment-gated fusion model
SAR	Synthetic aperture radar
S1	Sentinel-1
S2	Sentinel-2
NDCI	Normalized-difference chlorophyll index
OOS	Out-of-sample
RF	Random forest
SVR	Support vector regression
RMSE	Root mean squared error
MAE	Mean absolute error
